# Emergence of Nanotechnology as a Powerful Cavalry against Triple-Negative Breast Cancer (TNBC)

**DOI:** 10.3390/ph15050542

**Published:** 2022-04-27

**Authors:** Aiswarya Chaudhuri, Dulla Naveen Kumar, Deepa Dehari, Sanjay Singh, Pradeep Kumar, Pradeep Kumar Bolla, Dinesh Kumar, Ashish Kumar Agrawal

**Affiliations:** 1 Department of Pharmaceutical Engineering & Technology, Indian Institute of Technology (BHU), Varanasi 221005, India; aiswaryachaudhuri.rs.phe20@itbhu.ac.in (A.C.); dullanaveenkr.rs.phe20@itbhu.ac.in (D.N.K.); deepa.dehari.rs.phe18@itbhu.ac.in (D.D.); ssingh.phe@iitbhu.ac.in (S.S.); dinesh.phe@iitbhu.ac.in (D.K.); 2 Babasaheb Bhimrao Ambedkar University, Lucknow 226025, India; 3 Department of Pharmacy and Pharmacology, School of Therapeutic Sciences, Faculty of Health Sciences, University of the Witwatersrand, Johannesburg 2193, South Africa; pradeep.kumar@wits.ac.za; 4 Department of Biomedical Engineering, College of Engineering, The University of Texas at El Paso, 500 W. University Ave, El Paso, TX 79968, USA; bollaniper@gmail.com

**Keywords:** chemotherapy, nanoparticles, targeted therapy, triple-negative breast cancer (TNBC)

## Abstract

Triple-negative breast cancer (TNBC) is considered one of the un-manageable types of breast cancer, involving devoid of estrogen, progesterone, and human epidermal growth factor receptor 2 (HER 2) receptors. Due to their ability of recurrence and metastasis, the management of TNBC remains a mainstay challenge, despite the advancements in cancer therapies. Conventional chemotherapy remains the only treatment regimen against TNBC and suffers several limitations such as low bioavailability, systemic toxicity, less targetability, and multi-drug resistance. Although various targeted therapies have been introduced to manage the hardship of TNBC, they still experience certain limitations associated with the survival benefits. The current research thus aimed at developing and improving the strategies for effective therapy against TNBC. Such strategies involved the emergence of nanoparticles. Nanoparticles are designated as nanocavalries, loaded with various agents (drugs, genes, etc.) to battle the progression and metastasis of TNBC along with overcoming the limitations experienced by conventional chemotherapy and targeted therapy. This article documents the treatment regimens of TNBC along with their efficacy towards different subtypes of TNBC, and the various nanotechnologies employed to increase the therapeutic outcome of FDA-approved drug regimens.

## 1. Introduction

Breast cancer is the uncontrolled growth of breast tissue, especially of milk ducts and lobules. Cancer in milk ducts is called ductal carcinoma, comprising 80% of breast cancer cases, while in lobules, it is called lobular carcinoma which constitutes only 10% of the cases [1]. 

From the 2020 GLOBOCON cancer statistics, it was found that approximately 2 million women were diagnosed with breast cancer, out of which approximately 684,999 (6.9%) women deceased of breast cancer. Globally, after lung cancer, breast cancer is reported to be the second most diagnosed cancer [2]. Breast cancer is categorized based on the existence or lack of receptors, such as estrogen receptors (ERs), progesterone receptors (PRs), and the human epidermal growth factor receptor 2 (HER2/neu) [3]. Triple-negative breast cancer (TNBC) is devoid of the expression of the above-stated receptors [4] and is comprised of only 10–20% of total breast cancer cases [5]. TNBC is eminently invasive and shows enhanced metastasis in the brain and visceral organs, especially in the lungs. In one of the clinical studies, it was observed that after being diagnosed with TNBC, 46% of the patients developed distant metastasis, usually in the third year. Also, in comparison to other types of breast cancer, the patients suffering from TNBC exhibited a shorter survival time, with a fatality rate of 40% in the initial five years [6]. The clinical data further supplied the median survival time after metastasis and the recurrence rate after surgery, which were found to be 13.3 months and 25%, respectively [7]. The enhanced metastasis and recurrence contributed majorly to the low prognosis of TNBC. 

### 1.1. Epidemiology

Globally, it has been observed that approximately 2,088,849 women suffer from TNBC [8]. Epidemiologically, TNBC occurs mostly in African-American women (22.5–23.7%) of 40 years of age with menopause [9,10,11]. In one of the studies, it was observed that TNBC could also occur in women having a family history of BRCA1 mutated breast cancer [12]. In India, it was estimated that the incidence of TNBC was as high as 31% [13,14].

### 1.2. TNBC—Metastasis Driven Complexity

**Figure 1:**
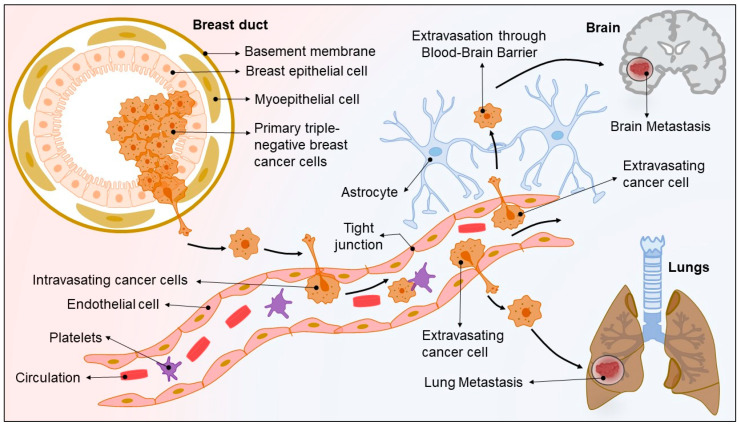
TNBC metastasis: Upon local invasion and intravasation, the TNBC cells experience various physiological changes like activation of EMT, MMPs, and pro-migratory signaling pathways, which enable the tumor cells to enter the circulation. After entering the systemic circulation, the tumor cells interact with platelets, which trigger the pro-survival pathways and restrict various apoptotic signaling pathways. The migrated tumor cells then extravasate via endothelial blood vessels and reach the secondary organs like the brain, bone, and lungs, where they get activated by metastasis-colonizing genes that allow them to enter a state of macrometastatic outgrowth [15].

TNBC metastasis is a complex process outlined by genetic as well as epigenetic transformation, stroma and tumor interactions, angiogenesis, intravasation via the basal membrane into the blood circulation or lymphatic circulation, and extravasation (Figure 1) [15]. It is explained that upon genetic and/or epigenetic transformation, the TNBC cells acquire self-renewal and migration ability, which aid them to invade the locally surrounded normal tissues and the circulation. During the local invasion and intravasation, the TNBC cells experience various phenomena like epithelial-to-mesenchymal transition (EMT), the disintegration of cell-cell junctions, overexpression of mesenchymal genes, and reduced expression of epithelial markers [16]. This entire process of migration and intravasation into the systemic circulation is triggered by several transcription factors, namely SLUG, SNAIL, TWIST, etc. [17,18,19]. In another study, it was observed that the expression of TGFβ (Transforming growth factor- β)/Smad signaling activates EMT, which in turn regulates WAVE3 (actin-binding protein belonging to the WASP/WAVE family), and the overall process leads to the TNBC cells intravasation. Likewise, triggering of C-X-C chemokine receptor type 4 (CXCR4) by C-X-C-Motif chemokine ligand 12 (CXCL12) induces the signaling of Mixed lineage protein kinase 3 (MLK3) and Extracellular signal-regulated protein kinases 1 and 2 (Erk1/2) proteins, which develop intravasation and lead to lung and bone metastases [20,21]. Moreover, it was found that when the Tropomyosin receptor kinase B (TRKB) receptor binds with brain-derived neurotrophic factor (BDNF), a metalloproteases network and calmodulin protein become regulated. As a result, the tumor—endothelial cell interaction gets altered, which leads to lung and brain metastasis in TNBC [22]. It is further noted that there are other genes like Epiregulin (EREG), Cyclo-oxygenase 2 (COX2), and Matrix metalloproteinases (MMP1, and MMP2) that are equally overexpressed in TNBC and play an important role in promoting lung metastasis [23].

The majority of the genes or proteins taking part in inducing TNBC metastasis have been observed to exhibit their respective roles during the early stages of TNBC, i.e., from migration to intravasation. This emphasizes the significant challenges faced during diagnosis and treatment in the early stage of TNBC. Therefore, identification of these TNBC metastasis-inducing genes and proteins provides an opportunity for the development of therapies against TNBC-metastasis. 

### 1.3. Heterogeneity of TNBC

Heterogeneity is the term used to describe the phenomenon where different cancer cells provide a distinct cellular morphology, gene profiling, proliferation potential, and metastasis. TNBC exhibits intra- as well as inter-tumor heterogeneity [24]. The heterogeneity exists due to various somatic mutations prevailing in the genes associated with TNBC. For understanding the heterogeneity of TNBC, The Cancer Genome Atlas (TCGA) Research Network scrutinized initial cancer cells through six platforms comprising DNA methylation, exome sequencing, microRNA sequencing, messenger RNA arrays, DNA copy number arrays, and reverse-phase protein arrays. By integrating obtained information from the six platforms, TCGA stated that the heterogeneity in TNBC mostly exists due to the mutation in genes related to DNA damage repair. This includes loss of *BRCA1*, *Tumor protein 53 (TP53)*, and *Retinoblastoma protein 1* (*RB1)* functioning [25]. Moreover, TCGA also noticed mutation in Phosphoinositide 3-kinases (PI3K)/Mammalian target of rapamycin (mTOR) signaling pathways due to the loss of lipid phosphatases PTEN (Phosphatase and Tensin homolog) or INPP4B (Inositol polyphosphate 4-phosphatase type II). However, heterogeneity becomes more substantiated when it was observed that some patients showed less mutation during diagnosis, while others showed rapid mutation [26]. It could thus be stated that due to such mutations within the TNBC cells, chemotherapy responds differently in different regions of a single tumor. The heterogeneity could also render resistance to the TNBC cells against chemotherapy [27]. 

Here, in the introduction segment, we have provided a slight insight into TNBC and discussed the epidemiology, complicated metastasis process, and the heterogeneity associated with the diagnosis of TNBC. This review further strives to highlight the various subtypes of TNBC, the experimental targets associated with its progression, the therapeutics used for its treatment, the various types of nanoparticles explored for handling its menace that may have some clinical application in near future as well as the study involving various ongoing clinical trials.

## 2. Subtypes of TNBC

TNBC is classified based on histology, gene-expression profiling, and genomic alterations [28]. However, tremendous advances have been made in explaining the gene-expression profiling for classifying TNBC, which paved the way for personalized treatment and guidance in clinical trials. 

### 2.1. Subtypes Based on Gene-Expression Profiling

**Figure 2:**
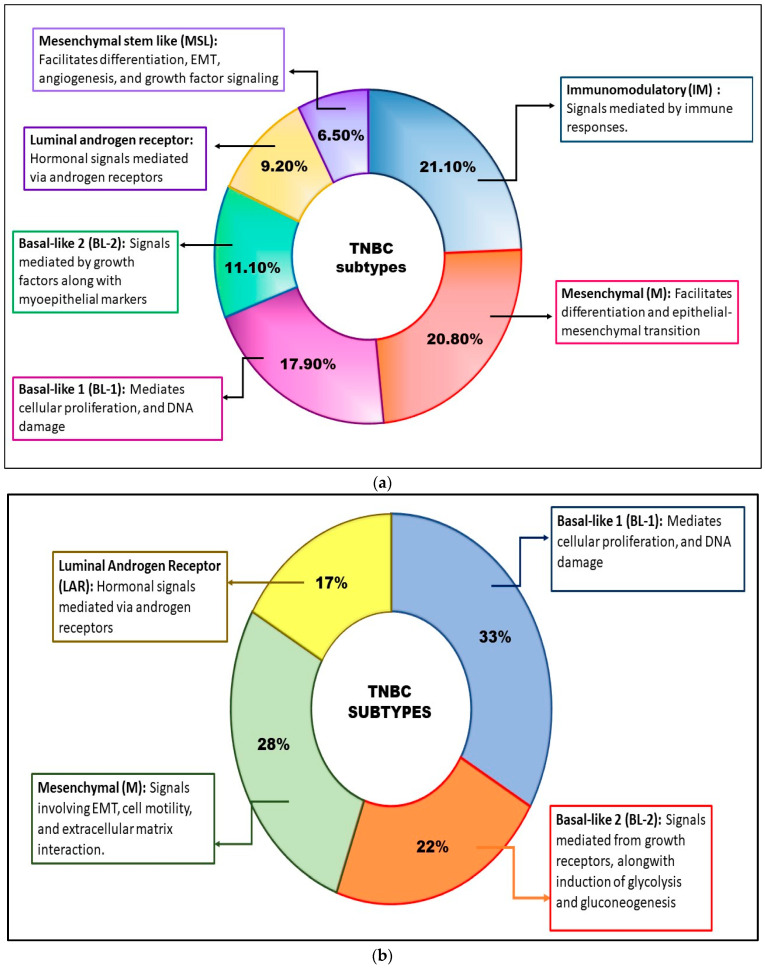
(**a**) Categorization of TNBC by Lehmann et al., 2011: In 2011, Lehmann et al. categorized TNBC into six subtypes based on gene profiling: basal-like 1 (BL-1), basal-like 2 (BL-2), immunomodulatory (IM), mesenchymal (M), mesenchymal stem-like (MSL), and luminal androgen receptor (LAR) [29]. (**b**) Categorization of TNBC by Lehmann et al., 2016: In 2016, on further analysis, Lehmann et al. categorized TNBC into four subtypes: basal-like 1 (BL-1), basal-like 2 (BL-2), mesenchymal (M), and luminal androgen receptor (LAR) [34].

Lehmann et al., 2011, categorized 6 subtypes of TNBC based on the genomic profiling of cancer cells obtained from 587 TNBC patients. The TNBC subtypes comprised of basal-like 1 (BL-1), basal-like 2 (BL-2), mesenchymal (M), mesenchymal stem-like (MSL), immunomodulatory (IM), and luminal androgen receptor (LAR) (Figure 2a) [29]. 

BL1 is associated with the overexpression of genes taking part in cell division, like Aurora kinase and MYC [30], while BL2 is associated with upregulation of genes in growth factor signaling like Epidermal growth factor receptor (EGFR), Wingless/Integrated (Wnt), Mesenchymal epithelial transition (MET), Insulin-like growth factor 1 receptor (IGF-1R), etc. [31]. Like the BL2 subtype, the MSL subtype also exhibited increased expression of genes associated with the signaling of growth factors. On the other hand, the M subtype is associated with overexpressed genes related to cell motility and differentiation [32]. IM subtypes are comprised of increased expression of genes associated with immune responses like cytokines signaling, antigen processing and signaling immune transduction pathways, etc. [33]. Lastly, the LAR subtype exhibited overexpression of genes associated with the metabolism of androgen and synthesis of steroids [32]. In 2016, Lehmann and his associates did a follow-up study on the TNBC classification and found that the IM and MSL subtype of TNBC was not associated with gene profiling; instead, the IM subtype was associated with tumor-infiltrating lymphocytes (TILs), and MSL was contributed to the tumor-associated stromal cells. Hence, they finally amended the classification, which led to four subtypes based on gene profiling, namely, BL1, BL2, M, and LAR (Figure 2b) [34].

On a similar note, Jiang et al., 2019, scrutinized 465 patients suffering from TNBC at Fudan University Shanghai Cancer Centre (FUSCC) based on clinical, genomic, and transcriptomic data, which lead to classify TNBC into four mRNA-based subtypes: (a) LAR subtype, associated with the signaling of androgen receptor, (b) IM subtype, involving immune cell and cytokine pathway signaling, (c) basal-like immune-suppressed (BLIS) subtype associated with DNA repair mechanism activation as well as immune response genes downregulation, and lastly, (iv) mesenchymal-like (MES) subtype involved with cell motility and differentiation [35]. 

#### 2.1.1. BL Subtypes TNBC

BL subtypes are considered the predominant TNBC subtypes. As mentioned earlier (Section 2.1), BL1 is associated with aberrant upregulation of genes associated with regulation of cell cycle and repairing of DNA damage like AKT2, BRCA2, CCND1, CDKN2A/B (cyclin-dependent kinase inhibitor 2A and B), CDK6 (cell division protein kinase 6), CHEK1 (checkpoint kinase 1), IGF1R, KRAS, MDM2 (mouse double minute 2 homolog), MYC, PIK3CA, PTEN, RAD51, RB1, and TP53, while BL2 is associated with abnormal regulation of genes related with the signaling of growth factors like EGFR, MET, IGF-1R, NGF (nerve growth factor), and Wnt/β-catenin pathways. It was thus suggested that the possible therapeutics for patients suffering from BL1 subtype TNBC includes inhibitors of poly (ADP-ribose) polymerase (PARP) and cytotoxic agents, while the patients suffering from BL 2 subtype TNBC need the inhibitors of PI3K/AKT/mTOR pathways and growth factors signaling. It was also reported that the patients suffering from BL1 subtype TNBC were found to be sensitive to cisplatin [36]. The proliferative nature of the BL1 subtype was supported by the increased expression of Ki67 mRNA and staining of nuclear Ki67 via immunohistochemistry (>70%, as compared to other subtypes ≈ 42%), which suggests that taxane-based chemotherapy is the most applicable class of chemotherapeutics for such a subtype [29]. It was observed that both BL subtypes showed an increased rate of pathologic complete response (pCR = 63%; *p =* 0.042) with taxane chemotherapy than with MSL (31%) or LAR (14%) subtypes [30]. Also, BRCA1 and BRCA2- mutant TNBC are regarded as basal-like subtypes as the gene expression patterns of these mutations correlate with the basal-like subtype [37].

#### 2.1.2. IM Subtype TNBC 

IM subtype is enriched with genes associated with immune responses and signal-transducing pathways like B cell receptor signaling pathway, dendritic cell (DC) pathway, interleukins (IL-7, IL-12), JAK (Janus kinase), NF-kB (nuclear factor kappa light chain enhancer of activated B cells), NK (natural killer) cell, TNF (tumor necrosis factor), Th1/Th2 (Type 1 and 2 helper) pathway, and T cell receptor signaling pathways. It was observed that the IM subtype is quite familiar with the medullary type of breast cancer due to the overlapping of the genetic signatures involved in their progression [33]. It is also worth mentioning that the IM subtype is associated with increased levels of immune cell infiltration, and as TILs are indicative of neoadjuvant chemotherapy response, such subtypes resulted in an effective clinical outcome [38,39,40,41]. Patients suffering from the IM subtype of TNBC are recommended with chemotherapies and immunotherapies [36].

#### 2.1.3. M Subtype TNBC

M subtype TNBC is associated with the abnormal expression of genes and signaling of pathways taking place during cell motility, interaction with the extracellular receptor, differentiation like TGF-β signaling, Wnt pathway, anaplastic lymphoma kinase pathway, etc., and regulation of cancer stem cells [36,42]. It was reported that the M subtype possesses sarcoma-like features and is hence susceptible to develop resistance towards chemotherapeutics. Thus, it was recommended that the patients suffering from M subtype TNBC should be treated with inhibitors of PI3K/AKT/mTOR, Src inhibitors, or drugs targeting EMT [32,43].

#### 2.1.4. MSL Subtype TNBC

The MSL subtype is quite familiar to the M subtype with some uniqueness in itself. Like the M subtype, MSL also involves aberrant expression of genes and signaling pathways in cellular motility, differentiation as well as cell-extracellular receptor interaction—but in low levels. The uniqueness of MSL lies with its involvement in the abnormal expression of genes and factors associated with growth factor signaling like signaling of EGFR, PDGFR (platelet-derived growth factor receptor), and GPCRs (G-protein coupled receptors), metabolism of inositol phosphate, signaling of ABC (ATP-binding cassette) transporter, adipocytokine, calcium, and ERK1/2 protein [32,42]. In addition to this, MSL also shows high expression of stemness-related genes, HOX genes as well as mesenchymal stem cell-specific markers. The stemness-related genes include ABCA8, ABCB1, ALDHA1 (aldehyde dehydrogenase 1 family, member A1), BCL2 (B-cell lymphoma 2), BMP2 (bone morphogenetic protein 2), ENG, PROCR (protein C receptor), and THY. The HOX genes include HOXA5, HOXA10, MEIS1, MEIS2, MEOX1, MEOX2, and MSX1, and mesenchymal stem cell-specific markers involves BMP2, ENG, ITGAV (integrin subunit alpha V), NGFR, NT5E (5′-nucleotidase Ecto), PDGFR, THY1, and VCAM1 (Vascular cell adhesion molecule 1). Like the M subtype, the patients with MSL subtype are recommended to procure PI3K/mTOR, and Src inhibitors (dasatinib), and antiangiogenic drugs [36]. In 2015, Burstein et al. conducted a study and showed that the MSL subtype also expresses genes that are exclusive to adipocytes like ADIPOQ, and PLIN1(perilipin), osteocytes namely OGN (osteoglycin), and IGF-1 growth factor [42].

#### 2.1.5. LAR Subtype

LAR subtype is regarded as the divergent subtype of TNBC, identified by the signaling of androgen receptor signaling and expression of luminal genes [34]. Although they are considered ER (ER α) negative, they are highly involved with pathways regulated by hormones, namely synthesis of steroids, metabolism of porphyrin, and androgen/estrogen as well as showing a low-level expression of ER, ErbB4, and prolactin [42]. It is worth mentioning that in this subtype of TNBC, androgen receptor (AR) is overexpressed with nine times more mRNA level, as compared to any other TNBC subtypes [29]. Further study on the gene profiling of LAR subtype revealed that LAR exhibits ESR1 expression (the gene required for Erα encoding), as well as expression of other estrogen-regulated genes including FOXA, GATA3, PG-R, and XBP1 (X-Box binding protein 1) [42]. In the immunohistochemistry study of the LAR subtype, an increased expression of AR, along with its downstream metabolic markers, the associated auxiliary activators are detected, which include ALCAM (activated leukocyte cell adhesion molecule), APOD (Apolipoprotein D), CLDN8, DHCR24 (24-dehydrocholesterol reductase), FKBP5, and PIP (prolactin-induced protein). Hence, the patients suffering from LAR subtype TNBC are recommended with anti-AR therapy [44], and HSP90 (heat shock protein 90) inhibitors [29]. 

#### 2.1.6. BLIS Subtype TNBC

BLIS subtype TNBC is associated with abnormal gene expression involving cell division, DNA replication, and DNA repair along with immune responses [42]. The immune responses involved in BLIS include the signaling pathway of the T-cell receptor, activation of B-cell receptor, complement cascades, and induction of DC cell chemotaxis [45]. It was observed that the BLIS subtype showed enhanced upregulation of proliferation-related genes like CENPF (centromere protein F), BUB1 (budding uninhibited by benzimidazoles 1), and PRC1 (protein-regulator of cytokinesis-1) and are hence considered as the highly proliferative type. This was further supported by a study that showed poor RFS (recurrence-free survival) and higher recurrence risk associated with BLIS [42].

### 2.2. Subtypes Based on the Histology of TNBC Cells

The phenotype of TNBC provides a distinct set of histologic classes. It was observed that histologically, TNBC is classified as invasive ductal carcinoma, medullary, metaplastic, apocrine, and adenoid cystic cancer. The metaplastic type TNBC is characterized by the area indicated by differentiation to a mesenchymal phenotype, while the medullary type TNBC is indicated by extensive lymphocytic infiltration. However, it was observed that more than 80% of TNBC belongs to the invasive ductal carcinoma type. Further, it was mentioned that these histologically-based variants are included in the TNBC datasets, which will contribute to the clinical and biologic heterogeneity of this group [28].

### 2.3. Subtypes Based on the Tumor Microenvironment (TME)

It has been expressed that the tumor microenvironment (TME) is also recognized as an indicator of immunotherapy efficacy as well as patient prognosis [39,46]. Further, the phenotype of TME gets characterized by the prevalence of TILs, which were found to be in high levels in patients suffering from early-stage TNBC [47,48]. It was observed that the presence of TILs in TNBC indicates enhanced overall survival, increased metastasis-free survival, as well as limited distant recurrence [49]. Based on these findings, Xiao et al., 2019, classified TNBC into three subtypes as per the plethora of microenvironment cells around tumor site and TILs: (i) immune-desert TNBC, having less microenvironment cell infiltrations, (ii) innate-immune activated TNBC, involving resting innate immune and non-immune stromal infiltrating cells, and (iii) immune-inflamed TNBC, having immune cells infiltrations of both adaptive as well as the innate type [50]. 

## 3. Potential Therapeutic Targets for TNBC Therapy

As mentioned previously (Section 1.3), TNBC exhibits high heterogeneity, which is the major limitation of chemotherapy. Also, TNBC is regarded as an aggressive type of cancer that grows faster and metastasizes to the brain and visceral organs, providing a much shorter average survival time of about 12 months to patients suffering from advanced TNBC. Therefore, the recognition of definitive targets, for providing efficient treatment against TNBC, becomes a noteworthy task. 

**Figure 3:**
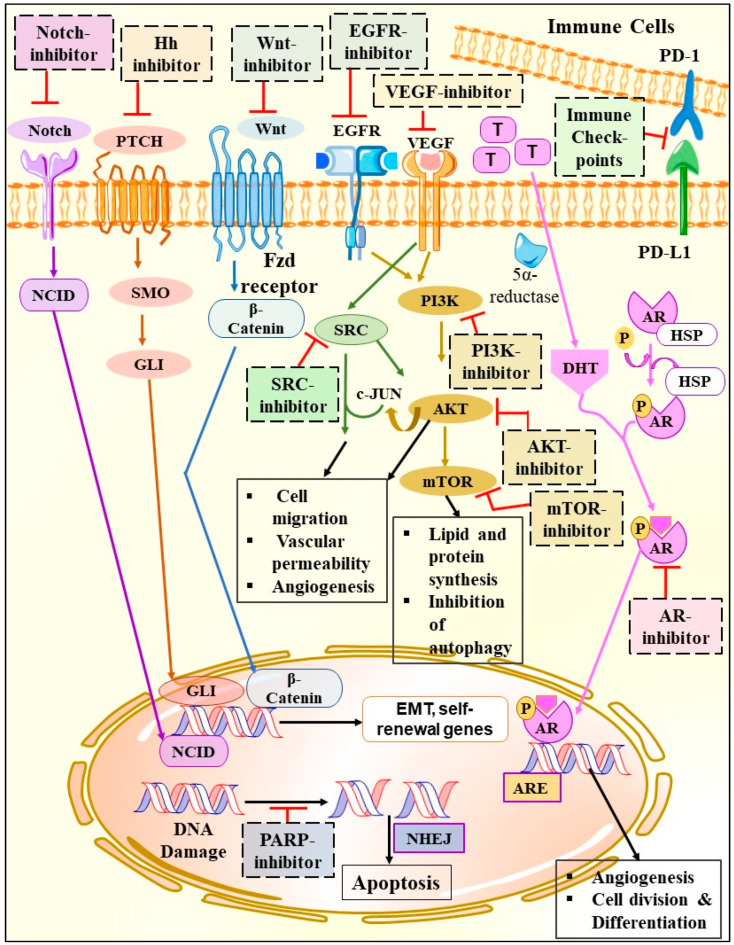
Schematic illustration of the potential targets and potential therapeutic agents, focused on the effective treatment of TNBC: Various signaling pathways have been involved in the etiology of TNBC, which encompasses EGFR, VEGFR, PDGFR, FGFR, Wnt, Notch, Hedgehog, immune checkpoint receptors, PI3k/AKT/mTOR, RAS, and JAK/STAT. Now, taking into account the intra- and inter heterogeneity, and the prospect of feedback mechanism to promote resistance to therapy, targeted inhibition is employed in combination with mitotic inhibitors and DNA damaging agents, which includes inhibitors of CDK4/6, CHK1/2, and PARP1. The red line indicates inhibition, and the black arrow indicates stimulation [51].

In the following section, we have concentrated on the available potential targets of TNBC, which includes some signaling pathways like angiogenesis, Hedgehog (Hh), Notch, Wnt/β-catenin, PI3k/AKT/mTOR, TGF-β, and Src kinase signaling pathways, and some specific receptors like EGFR, IGF 1R, PARP1 targeted receptors, programmed cell death ligand 1 pathway (PDL-1) targeted receptors, chondroitin sulfate proteoglycan 4 (CSPG4) protein targeted receptors, cytotoxic T lymphocyte antigen 4 targeted receptors (CTLA-4), and androgen receptors (AR) (Figure 3). 

### 3.1. Notch Signaling Pathways

The Notch signaling pathway is considered an exceptionally preserved targeted pathway that involves juxtracrine (cell-to-cell) communication, thus regulating several critical cellular processes [51]. Notch signaling is known for regulating self-renewal as well as differentiation processes, required for normal development of the mammary gland [52,53]. It was observed that the signaling pathway is triggered when the Notch ligand interacts with the Notch receptor, situated on the adjoining cell. Up till now, four Notch receptors and five Notch ligands have been identified. The five Notch ligands identified are Delta-like (DII)-1, 3, and 4, and Jagged (JAG)-1, and 2. Structurally, it was observed that all the Notch ligands are transmembrane proteins, having an extracellular DSL domain and multiple EGF-like repeats. The extracellular DSL domain is responsible for mediating the binding process between the Notch receptor and Notch ligands. In addition to this, the JAG ligand consists of an extra domain enriched with cysteine, which is absent in the DII ligand [54]. It was observed that a Notch ligand-receptor complex was formed when the Notch ligand binds with the Notch receptor. The binding procedure is facilitated by two proteolytic enzymes, i.e., ADAM (disintegrin metalloprotease) and TACE (TNF-α converting enzymes), which further aids in releasing the ectodomain of the Notch receptor [55,56]. The proteolytic activity then converts the Notch ligand-receptor complex into NEXT (Notch extracellular truncation). Finally, the NEXT is broken down via γ-secretase, and the Notch intracellular domain (NICD) get discharged, which then translocates from the cytoplasm to the nucleus [57], where it binds with the transcriptional activator, namely CSL complex (CBF1, RBPJK/Su(H)/LAG1, and Mastermind (MAML1-3, and MED3)) [58,59]. The binding then orchestrates CSL-complex activation, leading to transcription of downstream targets, which includes genes like ER, Hes, Hey, and VEGFR3 (vascular endothelial growth factor receptor 3), transcriptional factors like NF-κB2 and c-Myc, cell cycle regulators (cyclin D1 and p21), growth factor receptors, and angiogenesis and apoptosis modulators [51,60]. It was observed that dysregulation of the Notch signaling pathway often leads to aberrant self-renewal and differentiation of stem cells, which results in carcinogenesis [61,62]. 

In a study by Speiser et al., 2012, it was found that the progression of TNBC was associated with increased expression of Notch 1 and Notch 4 receptors [63]. The abnormal activation of NOTCH receptors results in aberrant controlling of the transcription of several oncogenes as well as tumor suppressor genes [64], which include CYCLIN D1, c-MYC, PTEN, and the BCL-2 pro-survival proteins [65,66,67]. Loss or reduced expression of NICD, NUMB (a negative regulator of EMT) also leads to TNBCs in association with poor clinical outcomes [68,69]. In 1987, Gallahan et al. inserted a mutagenic mouse mammary tumor virus (MMTV), which then generated truncated and active Notch1 and 4 receptors, resulting in the formation of breast cancer in mice [70]. On a similar trait, in 2013, Reipas and his associates revealed that p90 ribosomal S6 kinase, an oncogenic transcription factor is required for the growth of TNBC, which was found to be an activator of the Notch4 signaling pathway [71]. Thus, there exists strong evidence that indicates the involvement of the NOTCH signaling pathway, especially NOTCH1/NOTCH4 into the etiology of TNBC. Hence, targeting the Notch signaling pathway should provide a promising treatment platform against TNBC.

### 3.2. Hedgehog (Hh) Signaling Pathway

Hh signaling is also considered a preserved pathway, just like notch pathways. Hh signaling acts as a key signaling cascade in the proper development of the embryonic mammary gland as well as ductal morphogenesis. Hh signaling is also known to take part in EMT [72]. Hh signaling pathway is comprised of three ligands, namely Sonic Hedgehog (Shh), Desert Hedgehog (DHh), and Indian Hedgehog (IHh), where Sonic Hedgehog was regarded as the most targeted, one transmembrane receptor, PTCH, and one co-receptor, SMO. It was observed that the co-receptor SMO is required for the functioning of the Hh signaling pathway, but it has been inhibited by PTCH. So, the binding of the Hh ligand with the PTCH receptor led to its inactivation, which indirectly activates the SMO co-receptor. The activated SMO leads to the formation of a multiprotein complex, GLI complex, considered a hallmark in Hh pathway activation. Further, the functioning of the GLI complex is mediated by transcription factors GLI1, GLI2 (activator of the complex), and GLI3 (inhibitor of the complex). The activated GLI complex gets translocated in the nucleus, where the GLI1 and GLI2 transcription factor gets upregulated and engages itself in the transcription process, causing metastasis, angiogenesis, and apoptosis, resulting in the development of TNBC [73]. The transcription process also enhances the expression of proteins responsible for metastasis (SNAIL), and angiogenesis (angiopoietin-1, and 2) [74,75]. Moreover, SMO directly activates MYCN, which elevates the expression of transcription factors, FOXM 1 and cyclin D, leading to the proliferation of TNBC cells [76]. Furthermore, it was observed that the other transcription factors, namely NF-kB, FOXC1, and Hypoxia-induced factor (HIF)-1α are also involved in the deregulation of Hh signaling, along with TGF-β, RAS/MAPK (mitogen-activated protein kinases) signaling pathways, and the extracellular matrix protein osteopontin (OPN), thus, overall contributing to enhanced growth and invasion of TNBC [73]. It was reported by Mukherjee et al., 2006, that 70% of ductal carcinomas and 30% of metastatic breast cancer showed an overexpression of SMO receptors [77]. 

### 3.3. Wnt/β-Catenin Pathway

The Wnt signaling pathway serves a vital role in the patterning of embryonic tissue, migration of cells as well as its adhesion, maintenance of stem cells, and mediating epithelial-mesenchymal interactions [60]. This signaling pathway activates when Wnt proteins bind with LRP5/6 protein (LDL receptor-related protein5/6), and Frizzled protein (FZD; seven-pass transmembrane receptor protein) [78]. In absence of Wnt proteins/ligands, β-catenin is concealed within a complex comprising of axin, adenomatous polyposis coli (APC) tumor suppressor, glycogen synthase kinase-3β (GSK3β), and casein kinase 1 (CK1), which later triggers phosphorylation of β-catenin via CK1 and GSK3β and results in ubiquitination. This ubiquitination then compels the 26S proteasome to degrade β-catenin [79]. However, when Wnt proteins interact with LRP5/6 and FZD, they form a complex called the Wnt-LRP5/6-FZD complex, which inhibits GSK3β and leads to the cytosolic β-catenin stabilization. The free β-catenin then translocates from the cytoplasm to the nucleus, where it associates with T-cell factor (TCF)/lymphoid enhancing factor (LEF) to activate the expression of various downstream targeted genes that are responsible for regulating cell growth, proliferation, and apoptosis, thus mediating initiation as well as the progression of TNBC [78,80]. In a study, the up-regulated expression of FZD and LRP5/6 in TNBC cells was also observed. Also, TNBC cells exhibit a transcriptional knockdown of FZD/LRP6 that establishes its restraining activity in vivo [78]. It was further generalized that the association of the Wnt/β-catenin pathway with the progression of TNBC is related either with a gain of nuclear β-catenin or loss of membranous β-catenin [81].

### 3.4. TGF-β Signaling Pathway

The TGF-β signaling pathway is a pathway involved in the growth of cells, their differentiation, homeostasis, as well as apoptosis. TGF-β cytokines are comprised of many members, out of which TGF-β1, encoded via TGF-β1 gene [82,83] has been announced to play an essential part in breast cancer stem cells (BCSC). It was observed that the TGF-β receptor 1 (TGFBR1) is overexpressed in BCSC [84]. On further study, it was found that TGF-β1 induces EMT in mammary cells, which results in tumor formation [85]. This was further evidenced by a study performed by Sendurai et al., 2008, which exhibited that the mammary stem cells indicated increased expression of TGF-β1, thereby increasing their ability of mammospheres formation along with EMT gene expression like N- and E- cadherin, Slug, and Snail, associated with TNBC progression. On a similar note, Michael et al., 2011, reported that the CSCs (cancer stem cells) formed by TGF-β/TNFα induced EMT, showed enhanced self-renewing capacity along with increased tumorigenicity and chemotherapeutics resistance [86]. Also, the TGF-β1 pathway was found to generate SMAD2/3 and SMAD4 expression, causing activity like the synthesis of protein, growth proliferation, metastasis, and angiogenesis [51]. Thus, it could be inferred that TGF-β signaling plays a crucial character in the activation of EMT and procuring stemness, hence this pathway has been suggested as a novel therapeutic approach against TNBCs.

### 3.5. PI3K/AKT/mTOR Signaling Pathway 

PI3Ks are considered an important molecule of the PI3K/AKT/mTOR signaling cascade that lead to the growth of tumor cells. PI3Ks are heterodimers composed of p85 (regulatory subunit), and p110 (catalytic subunit). There are four PI3Ks isoforms currently known, namely α, β, ϒ, and δ [87]. PI3K/AKT/mTOR signaling pathway gets activated when stimulated by tyrosine kinases receptor, which further causes activation of PI3K, followed by AKT and mTORC1 phosphorylation [88]. The mTOR is a kinase protein composed of serine/threonine, responsible for controlling cellular proliferation, cellular growth, motility, and survival, as well as protein synthesis and transcription [89,90]. mTOR is comprised of two complexes, namely mTORC1 and mTORC2. It was found that both mTORC1 and mTORC2 induce S-phase kinase association protein, which leads to the synthesis of protein, growth, and proliferation of cells along with metastasis and angiogenesis. Hence, the progression of TNBC is found to be associated with the deregulation of the mTOR pathway [51]. It was further observed from the studies that in TNBC, the actuation of the PI3K/AKT/mTOR signaling pathway was also mediated via overexpression of EGFR (upstream regulators), and proline-rich inositol polyphosphatase (downstream regulator), mutation of the *PIK3Cα*, and loss of expression of *PTEN* [91,92,93]. Further, it was observed that the inactivation of p53 protein unleashes various tumorigenic pathways like FGFR (fibroblast growth factor receptor), cMET, and RAF (rapidly accelerated fibrosarcoma) kinases, which then activates the PI3K/AKT/mTOR signaling pathway [94]. However, TNBC progression was found to be rare when termed with the mutation of other downstream regulators of PI3K/AKT/mTOR signaling pathway (AkT, and mTOR) as well as cognate pathway (RAS, and MAPK) [95]. Thus, it could be inferred that the PI3K/AKT/mTOR signaling pathway can be used as a potential target of TNBCs.

### 3.6. EGFR 

EGFR is a transmembrane tyrosine kinase receptor, belonging to the family of HER/erythroblastosis virus oncogene B (ErbB) [96,97]. EGFR is responsible for regulating cell proliferation, cell differentiation, cellular invasion along with angiogenesis and apoptosis. Also, EGFR regulates the expression of Akt (PKB) and MAPK, responsible for inducing drug resistance [98,99]. Initially, EGFR was regarded as a target of lung cancer only, however, through recent studies it was revealed that EGFR also plays a role in the progression of TNBC [99]. It was revealed in a study that unlike the mutation of EGFR in the case of lung cancer, the progression of TNBC is related to an increased number of EGFR genes, and not the mutation of EGFR [100]. EGFR-overexpressing TNBCs were regarded as basal-like high-grade carcinomas. On the binding of BRCA1 to the miR-146a promoter, there occurs an increase in miR-146a transcriptional levels, which allows the binding of miR-146a to the 3’UTR of EGFR for promoting the degradation of its mRNA. As the TNBC is related to the EGFR gene number, it could be stated that the deficiency of BRCA1 and miR-146a prevents the degradation of mRNA, which in turn increases the number of gene expressions of EGFR and p-EGFR (Y1068) [101,102]. It was found that approximately 36% to 89% of TNBC showed an overexpression of EGFR. Further, it was observed that the survival of disease-free patients of TNBC patients is negatively associated with the overexpression of the EGFR gene [100,102,103]. Hence, it is reflected that EGFR can serve as a potential target for TNBC therapy.

### 3.7. IGF1R

In a clinical study, it was found that 50–75% of TNBCs showed enhanced expression of the IGF1R. IGF1R causes growth, invasion, as well as metastasis in patients suffering from TNBC. It has been reported that IGF1R increases the metastasis in cancer cells by inducing anchorage-independent growth, which became evident from a pre-clinical trial that showed an over-expression of IGF1R in the tumor initiation site as well as in the site where metastasis took place. Further, it was observed that IGF1R also inhibits the apoptosis caused due to the administration of the chemotherapeutic drug, hence suggesting an incidence of chemo-resistance [104].

### 3.8. PARP1

PARP1 belongs to the class of DNA repair enzymes, and plays a vital character in managing genomic stability, DNA repairing, regulating the progression of the cell cycle, and apoptosis [105]. PARP1 responds to single-stranded DNA damage via various repairing mechanisms like base excision repair mechanism, nucleotide excision repair mechanism, or mismatch repair mechanism and hence maintains the genomic integrity [106]. Hence, it could be stated that PARP1 inhibition causes loss of DNA repair functioning, inducing apoptosis [107].

On a similar note, it was observed that in the absence of the PARP1 enzyme, an aggregation of single-strand breaks (ssDNA) occurs, resulting in the generation of double-strand breaks (dsDNA), which were then repaired by homologous recombination (HR). HR is an error-free repairing process involving BRAC1 and BRAC2 proteins. So, the presence of PARP1 inhibits the functioning of HR, causing mutations in BRAC proteins [108], and from various studies, it was found that ≈70% of mutated BRCA1 and ≈16–23% of mutated BRCA2 breast cancers are regarded as TNBCs [109]. Therefore, inhibition of the PARP1 enzyme can also be used as a target in TNBC therapy.

### 3.9. Src Kinases

Src is a tyrosine kinase protein of the non-receptor type that belongs to the Src family kinases (SFKs). Src gets activated by two means, either via cytoplasmic proteins like focal adhesion kinase (FAK), Crk-associated substrate (CAS), playing a distinct role in integrin signaling, or via activation of ligand belonging to cell-surface receptors like EGFR, FGFR, VEGFR, etc. It was observed that SRCs regulate various signal transduction pathways associated with cell adhesion, cell migration, invasion, and angiogenesis [110]. In a study, it was found that TNBC is associated with overexpression of c-SRC kinases, also known as proto-oncogene tyrosine-protein kinase Src. The overexpression of c-Src in TNBC is responsible for tumorigenic proliferation, migration, and invasion [111,112]. Also, c-Src overexpression facilitates bone metastases in the case of metastatic TNBC. It was found that the increased activity of Src kinase is either due to its increased transcription or due to its deregulation caused by the overexpression of the upstream growth factor receptors including EGFR, PDGFR, FGFR, VEGFR, integrin, FAK, etc. [110]. 

### 3.10. Immune-System Targeting

Immune checkpoint inhibitors like anti-PD-1, anti-PD-L1, and anti-CTLA-4 demonstrate a novel type of immunotherapy for the treatment of cancer. It was found that in cancer progression, the immune response gets compromised. Research evidence revealed that T-lymphocytes are responsible for activating the distinct immune responses against an emerging antigen. Further, it was observed that lymphocyte surface receptors get stimulated when interacting with an antigen-presenting cell (APC) [113]. Cell activation needs definite identification of the presented antigen, as well as a specific signal from co-stimulators that are mobilized during the generation of the immune synapse. Such cell effector functions are inhibited by signals produced by negative cell receptors. The stated mechanism is anticipated to prevent the undesirable effects of overstimulation, causing an autoreactive response or stimulation of carcinogenesis once the defensive role of the lymphocyte antigen is swept. PD-1 (CD279) belongs to such type of negative receptor. Similar to PD-1, CTLA-4 is also a negative receptor available on APC, which on binding with CD80-B7-1, and CD86-B7-2 ligands activates an inhibitory reaction, which suppresses the immune responses, blocks the responses of T-lymphocytes, decreases the T-lymphocyte proliferation, and limits the secretion of cytokine. All of these contribute to an immune deficiency in cancer patients [114]. 

Hence, it was suggested that immunotherapy deals with the unblocking of the suppressed immune system and activating the functioning of T-lymphocyte within the lymph node, which gets translated to an effective immune response against TNBC.

#### 3.10.1. PD-L1

Programmed cell death (PD-1) receptor along with its ligand PD-L1 have been identified as biomarkers, because they are overexpressed in TNBC more than in any other type of breast cancer [115,116]. The concept of immunotherapy lies in the fact that sometimes cancerous cells escape recognition as well as avoid destruction from the host immune system because of the immune checkpoint system, which provides ample opportunities for the cancerous cells to grow, migrate, invade, proliferate, and metastasize. In such cases, immunotherapy plays a vital part in blocking the immune checkpoint system, thus providing a therapeutic and effective antitumor immunity [117].

PD-1 is an inhibitory receptor belonging to the family of B7-CD28. PD-1 is overexpressed on the activated lymphocytes, non-lymphocytic cells like activated monocytes and dendritic cells, B-cells, and natural killer cells [118]. PD-1 is comprised of two ligands, PD-L1, and PD-L2, out of which PD-L1 is overexpressed in cancer cells, tumor-infiltrating lymphocytes, fibroblasts, and macrophages [118,119]. In one of the studies, it was observed that statistically, about 45% of patients suffering from TNBC showed enhanced upregulation of both PD-L1 and PD-1, while 59% showed overexpression of PD-L1, and 70% showed overexpression of PD-1 [120,121]. It was observed that the interaction of PD-1 to PD-L1, made the T-cells less active and form an inhibitory state, which provides a reduced immune response towards foreign antigens. This mechanism proved profitable to normal cells in preserving an immune balance and preventing an autoimmune response. However, this mechanism can lead to the detection of tumor cell evasion and elimination induced by the immune system [122]. Hence, it could be indicated that the PD-L1 positivity can be considered a TNBC biomarker that could be targeted via immune checkpoint inhibitor for better therapeutic response [123].

#### 3.10.2. CTLA-4

CTLA-4 is an inhibitory receptor of T-cell, which is found to be overexpressed on activated CD8+ T cells [124]. Like PD-L, CTLA-4 also restricts the activation of T-cells by preventing its binding with its co-stimulatory molecules like CD80, and CD 86, resulting in detection of tumor cell evasion as well as its elimination by the immune system [125]. Hence, CTLA-4 also acts as a potential target against TNBC.

### 3.11. CSPG4 Proteins

CSPG4 is a proteoglycan, situated on the cell surface and found to be overexpressed in both melanoma cells and TNBC cells. It is also referred to as a melanoma-associated antigen or melanoma chondroitin sulfate proteoglycan [126]. CSPG4 protein spreads over the endothelial basement membrane, stabilizing the interaction between cell-substratum and resulting in events like cellular growth and proliferation, angiogenesis, and metastasis [127]. 

### 3.12. Androgen Receptor (AR)

ARs belong to the family of nuclear steroid hormone receptors [128]. The activation of AR is conducted by either the ERK-dependent pathway or the ERK-independent pathway. The ERK-dependent pathway includes the interaction of cytoplasmic AR with proteins like PI3K, Ras GTPase, and Src proteins, whereas the ERK-independent pathway involves phosphorylation of mTOR, activation of PKA, and inactivation of forkhead box protein O1 (FOXO1). Both the activation pathways ultimately result in cellular proliferation, EMT, angiogenesis, and metastasis [129,130]. It was observed in a clinical study that 25–75% of TNBC progression is due to overexpression of AR, mostly in the LAR subtype of TNBC [131,132]. In ASCO annual meeting, 2017, an investigation on AR expression in patients with TNBC showed that ≈30% of the patients were found to have positive AR expression [86]. Hence, it is contemplated that targeting AR may provide a potential platform for the treatment of TNBC.

Hence, from the above findings, it was observed that before providing proper treatment, one must identify the targets, which may be either proteins (CSPG4, Src Kinases), signaling pathways (Notch, hedgehog, Wnt-β, TGF-β, PI3K/AKT/mTOR), receptors (EGFR, IGF1R, AR, PD-1, CTLA-4) or enzymes (PARP1). It was observed that deregulation of these targets leads to TNBC progression. So, if one can identify those targets responsible for the progression, then their deregulation can be restricted or minimized, which will further reduce the risk of cancer growth. Moreover, based on the identifiable targets, various novel treatment options are under development. We have managed to assemble the current targets that have been proven to be promising for the treatment of TNBC. 

## 4. Available Drugs Used in TNBC Treatment 

### 4.1. Chemotherapy

Chemotherapy is considered as the main conventional therapeutic approach against TNBC because it is devoid of ER, PR, and HER2 expression, and as a result, the endocrine therapies remained ineffective against it [133]. Many clinical studies confirmed the therapeutic efficacy of the chemotherapeutic drugs in both neoadjuvant and adjuvant setups [134]. It was reported that according to the current standard of care (SOC), neoadjuvant and adjuvant chemotherapy was considered a key approach for the treatment of early TNBC [135]. From the clinical trials it was observed that with chemotherapy, the median progression-free survival (PFS) and median OS ranges from 1.7 to 3.7 months and 10 to 13 months from the onset of metastasis, respectively. Moreover, patients with advanced TNBC when treated with chemotherapy showed a median PFS and median OS of 4–6 months, and 11–17 months, respectively [136]. The chemotherapeutic agents include taxanes (anti-microtubule agents), anthracyclines (DNA intercalating agents and, topoisomerase inhibitors), cyclophosphamide (DNA alkylating agents), 5- fluorouracil (anti-metabolite), and platinum compounds [137]. 

#### 4.1.1. Taxanes

Taxanes are anti-mitotic agents that inhibit the depolymerization of microtubules, thereby ceasing the cell division at the prometaphase stage of the cell cycle [138,139]. The inhibition of depolymerization prevents the cell from forming spindles and spindle fibers, resulting in inhibition of cell division. In addition to this, the anti-cancer activity by taxanes was also performed via activated macrophages [133]. Commonly used taxanes include paclitaxel, nab-paclitaxel, and docetaxel [140]. From the gene profiling analysis, it was suggested that BL1 and BL2 subtypes of TNBC seemed to be sensitive to antimitotic drugs like paclitaxel, docetaxel, etc. Moreover, basal-like subtypes showed enhanced remission rates by four times as compared to MSL and LAR subtypes of TNBC [133]. 

#### 4.1.2. Anthracyclines 

Anthracyclines are the class of anticancer drugs that are derived from Streptomyces peucetius var. caesius [133]. Two theories were proposed to elucidate the mechanism of action of Anthracyclines against cancer. One of them is DNA intercalation, while the other one involves enzyme interaction. 

*DNA intercalation*: Anthracyclines are comprised of a chromophore moiety that is inserted between the adjacent base pairs of DNAs during its localization to the nucleus of the cell, thereby inhibiting DNA as well as RNA synthesis, and resulting in blocking the cell division. 

*Enzyme interaction*: Anthracyclines interact with topoisomerase II and form a complex that prevents the re-ligation of DNA breaks during DNA replication, thereby preventing cell division and promoting cell apoptosis. Various anthracyclines used for the treatment of TNBC are daunorubicin, doxorubicin, epirubicin, idarubicin, mitoxantrone, valrubicin, etoposide, and teniposide [141].

From the performed clinical trials, it was observed that the fatality rate was decreased by 38% and 20% in younger patients (<50 years), and elderly patients (50–69 years), respectively, when treated with anthracyclines [142]. However, for specific responses of chemotherapy, the combination of anthracyclines and taxanes was used widely among various subtypes of TNBC. It was observed that patients suffering from BL1 or MSL subtype TNBC showed an increased rate of pCR, as compared to BL2, and LAR subtypes, which are not sensitive to combination regimens [133]. 

#### 4.1.3. Cyclophosphamide 

Cyclophosphamide possesses no anticancer activity on its own, however, its metabolites, namely nitrogen mustard and acrolein, facilitates the inhibition of cell division via their alkylating properties. Cyclophosphamide, after entering the body, gets converted to aldophosphamide by microsomal oxidase, which further gets triggered by cytochrome P450 to generate the metabolites, which then prevent the linking of double-stranded DNA by adding an alkyl group to its guanine bases, causing breakage of the DNA strand and the inability of the cancer cell to divide, eventually facilitating the cancer cells to die. In recent times, a taxane and cyclophosphamide combination regimen has been employed as the optimum neoadjuvant chemotherapy for TNBC [143].

Wu et al., 2014, reported that the adjuvant chemotherapy regimen comprising of cyclophosphamide, methotrexate, and fluorouracil effectively limit the regional recurrence as well as prolong the disease-free survival of TNBC patients, especially those with a tumor diameter larger than 2 cm and that have undergone partial mastectomy [144]. Moreover, it was reported from the clinical trials that the BL1 subtype conceived increased pCR rate by 52% as compared to BL2 (0%), LAR (10%), and MSL (23%) [145]. 

#### 4.1.4. Antimetabolites 

Antimetabolites interfere with DNA synthesis and exert cytotoxic effects, and are hence considered a chemotherapeutic agent acting against TNBC. Antimetabolites are pyrimidine or purine analogues with an altered chemical group and which can induce cell apoptosis at the S phase of the cell cycle by inhibiting the enzymes required for the production of nucleic acid. Among antimetabolites, fluoropyrimidine, as represented by 5- fluorouracil (5-FU), and capecitabine are accepted and employed as first-line anticancer drugs against various types of cancer [146]. 

The mechanism of action of fluoropyrimidines involves inhibition of thymidylate synthase (TYMS). The fluoropyrimidines are cleaved into three different metabolites, namely fluorodeoxyuridine monophosphate (FdUMP), fluorodeoxyuridine triphosphate (FdUTP), and fluorouridine triphosphate (FUTP) [147]. FdUMP then forms a covalent bond with TYMS [148] and prevents the transformation of dUMP to deoxythymidine monophosphate (dTMP), which is considered essential for the synthesis of pyrimidine and DNA. The formation of covalent bonds also simultaneously blocks the transformation of tetrahydrofolate to dihydrofolate, an important element of the folate pathway, required for the synthesis of DNA as well as modification of DNA and RNA. As a result, it causes growth arrest and facilitates cell death [149]. Other antimetabolites administered for the treatment of TNBC include floxuridine, cytarabine, gemcitabine, decitabine, and vidaza. Li et al., 2015, reported from their clinical studies that the fluoropyrimidines in combination with platinum agents proved effective against patients suffering from metastatic TNBC, with acceptable side effects [150].

#### 4.1.5. Platinum Compounds

Platinum compounds are widely employed for the treatment of breast, colon, lung, and ovarian cancers. Platinum compounds are also considered as alkylating compounds that can bind with DNA and induce its breakage, and, as a result, inhibit cell repair and facilitate cell apoptosis. Cisplatin and carboplatin are widely used anticancer drugs [151].

Zhang et al., 2015, performed a clinical trial (NCT00601159) where the cisplatin was combined with gemcitabine (GP) as the first-line chemotherapeutics against metastatic TNBC. It was observed that the combinatorial regimen had an efficient activity with suitable safety, especially for the patients suffering from BL subtypes of TNBC [152,153]. Von Minckwitz conducted a clinical trial where carboplatin-based combined treatment was provided to random 269 TNBC patients, whereas non-carboplatin-based combined treatment was provided to 299 TNBC patients. It was observed that administration of carboplatin with taxanes and anthracyclines showed an increased pCR rate than other types of breast cancer [154].

**Figure 4:**
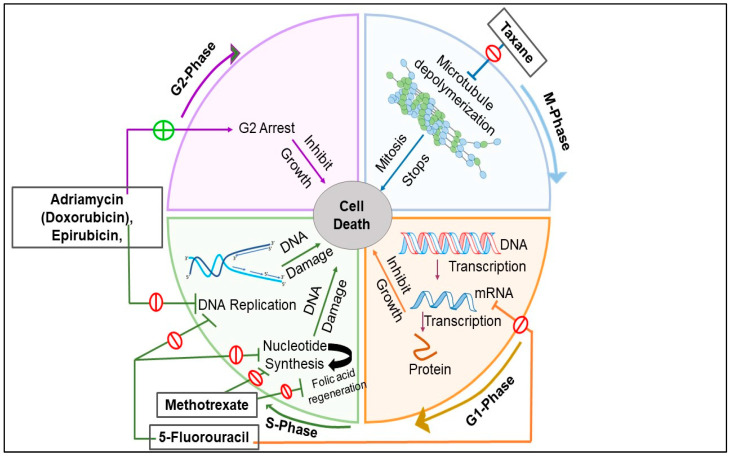
Mechanism of action of the combined drug regimen.

Based on various clinical trials and research studies, the national comprehensive cancer network guidelines recommended administration of combinatorial chemotherapeutics regimens for the treatment of TNBC like Taxane + Adriamycin + cyclophosphamide (TAC), Taxane + cyclophosphamide (TC), Adriamycin + Cyclophosphamide (AC), Cyclophosphamide + Methotrexate + Fluorouracil (CMF), Cyclophosphamide + Adriamycin + Fluorouracil (CAF), and Cyclophosphamide + Epirubicin + Fluorouracil + Taxane (CEF-T) [133]. Figure 4 describes the different mechanism of action of the combined drug regimen, revealing the essence of combination therapy.

However, it was observed that due to the existence of molecular heterogeneity in TNBC progression, it exhibits increased chemosensitivity along with a risk of early relapse. Hence many efforts are being made to improve the TNBC treatment for both responders and non-responders, which leads to the emergence of targeted therapy against TNBC [155]. 

### 4.2. Targeted Therapy 

Without a distinct and proper knowledge of the molecular pathogenesis of TNBC, it is difficult to develop any new effective targeted therapy for its treatment. However, in the last few years, various insights were procured regarding the aberrant signal transduction pathways that play a vital aspect in the TNBC progression, resulting in the evolution of targeted anticancer therapeutics (Figure 3). 

#### 4.2.1. γ-Secretase Inhibitors (GSIs) 

Targeting Notch signaling appeared as a therapeutic approach for patients suffering from TNBC, which includes GSIs and monoclonal antibodies (mAbs) [156]. GSIs prevent the cleavage of the NEXT complex (mentioned in Section 3.1) and prevent the release of active forms of Notch receptors, thereby inhibiting the transcriptional activity, which interferes with the cell cycle process, leading to apoptosis. Further, treatment with GSI causes upregulation of the pro-apoptotic protein, named Phorbol-12-myristate-13-acetate-induced protein 1 (NOXA), and restricts the formation of CSC colony, resulting in apoptosis of TNBC cell [157]. 

Several novel therapeutics involving GSI was administered in combination with chemotherapeutics to decrease the limitations associated with the monotherapy. RO-4929097 and MK0752 are the GSIs that are investigated in phase I and phase II clinical trials. Recently, it was disclosed that the combination of PF-03084014 (GSI) and docetaxel have surpassed the clinical trials, and are hence used as a therapeutic approach against TNBC [158]. In addition, a combination of another GSI (RO-4929097) with Paclitaxel and Carboplatin has been employed in phase I clinical trials [159]. The preliminary results exhibited the feasibility of the combinatorial approach of GSI and chemotherapy, promoting further studies regarding the combination approach of notch signaling inhibitors with chemotherapeutics against TNBC. However, GSI exhibits certain side effects like gastrointestinal impairment. Therefore, much work needs to be done for providing supportive effects after GSI treatments [156].

#### 4.2.2. PARP Inhibitors

As mentioned in Section 3.8, PARP is considered a damage recognition repair protein that initiates the repairing of single-strand break (SSB) via base excision repair. PARP inhibition causes accumulation of SSBs, leading to double-strand break (DSBs) formation. As reported, BRCA mutant cells cannot repair DSBs error-free and ultimately result in apoptosis [160]. Thus, PARP inhibitors could be considered as a potential platform that could be targeted for treating patients suffering from BRCA-mutated type TNBC [133]. Epidemiologically, 40% of TNBC patients exhibit BRCA1/2 mutations, and 60% of TNBC patients exhibit BRCA1-mutation [161]. Moreover, it was reported that the black and Hispanic populations have a probability of bearing BRCA1/2 mutations [162].

The inhibitors of PARP are nicotinamide mimetics that interact with the NAD+ site of PARP receptors reversibly, preventing their PARylation and subsequent DNA repairing. PARylation is prevented by ambushing PARP-1 on DNA, which further causes a configuration change and unbinds the DNA, resulting in the generation of stalled replication forks that collapse into lethal DSBs, causing apoptosis during the S-phase [163]. Hence it was indicated that trapped PARP—DNA complexes are considered more cytotoxic with increased anti-proliferative activity as compared to DNA polymerase, which is a PARP-dependent DNA-damage repair complex [164]. 

In recent times, many PARP inhibitors like olaparib, veliparib, rucaparib, niraparib, talazoparib (BMN673), etc., were developed, which are undergoing clinical trials [165]. So far, based on phase III clinical trials (OlympiAD and EMBRACA), olaparib and talazoparib have received approval from the FDA, and EMEA for their usage against BRCA1 and BRCA2 mutated TNBC patients as well as metastatic HER2- breast cancer patients [163]. According to the EMBRACA trial, talazoparib was compared with TPC regimen (capecitabine, eribulin, gemcitabine, or vinorelbine) in BRCA mutated TNBC patients. It was observed that talazoparib showed prolonged median PFS (8.6 months) in comparison to the control group (5.6 months). In addition, the objective response rate (ORR) was found to be enhanced in the talazoparib group (62.6%) in comparison to the TPC group (27.2%) (*p* < 0.001). The OlympiAD trial was also performed to compare the therapeutic efficiency of olaparib against TPC in BRCA mutated TNBC patients, and also in HER2-negative metastatic breast cancer (MBC) patients. It was observed that like talazoparib, the olaparib group also showed enhanced median PFS (7.0 months) in comparison to the TPC group (4.2 months) (*p* < 0.001) [161]. Recently, for increasing the sensitivity of PARP inhibitors towards BRCA mutated TNBC, PI3K inhibitors were co-administered to promote the homologous recombination deficiency, thereby downregulating the BRCA genes. Based on this finding, a phase I clinical trial (NCT01623349) is ongoing, which is comprised of an inhibitor of PI3K, named BKM120 (Novartis^®^), in combination with the inhibitor of PARP (olaparib) in metastatic TNBC patients [166].

#### 4.2.3. PI3K/AKT/mTOR Inhibitors

The PI3K/AKT/mTOR signaling pathway plays an essential part in promoting carcinogenesis and tumor growth in TNBC subtypes. PI3K pathway activation is associated with PTEN loss (35%) or PIK3CA mutation (7%), or both (30%). It was reported that 60% of TNBC is associated with low expression of PTEN proteins [167,168]. The mTOR downstream signaling promotes translocation of mRNA and phosphorylation of various substrates that accompany several anabolic processes taking part in TNBC progression [169]. Similarly, activation of AKT downstream signaling promotes cell growth, invasion, and migration in TNBC subtypes. The AKT1 mutation is also associated with loss of PTEN expression [161]. 

It was mentioned that there exist six different types of PI3K/AKT/mTOR pathway inhibitors, which include pan-class I, isoform-selective (PI3K blocker), rapamycin analogs, active-site mTOR blocker, Pan-PI3K/mTOR blocker, and AKT blockers. It was recommended that simultaneous targeting with more than one class of inhibitor seemed more beneficial than a single PI3K/AKT/mTOR pathway inhibitor [169].

So far, class I pan-PI3K blocker was found to be more widely used against TNBC than isoform-specific inhibitors. As observed from the phase III clinical study, Buparlisib (BKM120), an inhibitor/blocker of PI3K, showed an effective therapeutic efficacy against all isoforms of PI3K. However, for the treatment of TNBC, presently Buparlisib is under phase 2 trial (NCT01790932) [170]. On a similar note, the preclinical study of Alpelisib, another oral class I α-specific inhibitor of PI3K, showed a decreased AKT phosphorylation in different subtypes of TNBC, which includes BL-1, M, and MSL. A phase 1 clinical trial (NCT03207529) of Alpelisib in combination with enzalutamide is ongoing for the treatment of TNBC. Furthermore, rapamycin, an inhibitor of the mTOR pathway, showed significant inhibition of tumor growth by 77–99%, when compared with doxorubicin [88]. Ipatasertib, and Capivasertib (AZD5363). The highly selective oral AKT inhibitors target the phosphorylated AKT conformations and are used for the treatment of TNBC [168]. By tracking the clinical success of these drugs, many clinical trials were accomplished using AKT inhibitors against TNBC. LOTUS trial was executed to evaluate the safety and efficacy profile of the ipatasertib and paclitaxel combination, as compared to paclitaxel alone. It was observed that the patients who received ipatasertib and paclitaxel combination showed a prolonged median PFS of 6.2 months, whereas patients with only paclitaxel showed a limited median PFS of 4.9 months (*p* = 0.037). Similarly, a PAKT trial was also conducted to appraise the safety and efficacy profile of a capivasertib and paclitaxel combination in comparison to paclitaxel alone. The combination group showed a prolonged PFS of 5.9 months, while the paclitaxel group showed a PFS of 4.2 months (*p* = 0.06) [161]. However, it was observed that when the PI3K/AKT/mTOR signaling pathway is inhibited, it triggers a negative feedback loop that limits the efficacy of the inhibitors and promotes a resistance towards the single-agent receptor tyrosine kinase (RTK) inhibition. Further studies showed that AKT inhibition provokes FOXO-dependent transcription that activates RTKs, PI3K inhibition restricts AKT activation but at the same time enhances MAPK signaling, and mTOR inhibition upregulates RTKs resulting in rebound activation of AKT [168]. In this context, many preclinical studies were performed to overcome this problem, and finally, it was observed that the compounds focusing on different cognate molecules in the PI3K/AKT/mTOR pathway were combined, then a synergistic activity is offered that blocks the triggering mechanism of the negative feedback loop. Xu et al., 2013, reported that the combination of an inhibitor of the mTOR pathway (MK-8669) and an inhibitor of AKT pathway (MK-2206) showed an improved tumor-growth inhibition ratio, as compared to single agents (*p* < 0.001) [171]. Gedatolisib (mTOR and PI3K inhibitor) in combination with PTK7 antibody-drug conjugate (NCT03243331), showed an effective inhibition (IC_50_ 0.042 μmol/L) in the TNBC cell line. Similarly, Apitolisib (GDC-0980), which also inhibits both the mTOR and PI3K pathway, showed 50% tumor growth inhibition in the TNBC cell lines. Thereby, many preclinical studies on PI3K/AKT/mTOR inhibitors are performed as well as ongoing, however, a few received the opportunity to pass to the clinical trial for the treatment of TNBC [88].

#### 4.2.4. Growth Factor Inhibitors

A total of 60% of BL subtype TNBC is associated with the overexpression of EGFR. However, it was found that there are certain cases where BL2, and MSL subtypes TNBC are also associated with EGFR overexpression [172]. Such observations lead to the development of anti-EGFR targeted therapies [165]. Anti-EGFR targeted therapies include small molecular tyrosine kinase inhibitors (TKIs) and mAbs [109]. Numerous clinical trials were performed for assessing the efficacy and safety profiles of TKIs, but the results proved to be disappointing for both monotherapy as well as polytherapy with chemotherapeutics. A phase II clinical trial of Gefitinib and Erlotinib combination was performed in metastatic and recurrent breast cancer, which showed a partial response of 0–3% [173,174,175], while a phase I clinical trial of Erlotinib and Bendamustine combination in stage III and IV TNBC showed an ORR of 0% along with severe lymphopenia as an adverse effect [176]. In recent times, five clinical trials involving EGFR targeted TKI are ongoing in the United States, which includes TKI monotherapy, and TKI polytherapy in combination with chemotherapeutics, mTOR inhibitors, AMPK (AMP protein kinase) activators, and anti-VEGF mAb. Similarly, various clinical trials targeting anti-EGFR mAbs are conducted for evaluating their efficacy and safety against TNBC. So far, six-phase II clinical trials were performed [102]. A clinical trial using cetuximab, an anti-EGFR mAb as a monotherapy as well as a polytherapy with chemotherapeutics (Carboplatin) was conducted. It was observed that the ORR of the combination group was found to be 17%, while for the single-agent group it was found to be only 10%. Cetuximab binds to overexpressed EGFR in TNBC with a higher affinity, and, as a result, blocks the ligand-induced phosphorylation of EGFR [177]. Baselga et al., 2013, reported that in patients with advanced TNBC, the combination group of cisplatin-cetuximab showed a response rate of 20%, as compared to only 10% in the cisplatin group [102]. Currently, another human anti-EGFR mAb, panitumumab in combination with carboplatin and gemcitabine (NCT00894504) for the treatment of TNBC, is under investigation [165]. The efficacy of anti-EGFR mAbs was also investigated in operable TNBC patients. Overall, it was observed from various clinical trials that for the treatment of EGFR overexpressed TNBC, anti-EGFR mAbs seemed to be a slightly better therapeutic option than that of EGFR TKIs [102].

#### 4.2.5. Src Inhibitors

Src is a non-receptor tyrosine kinase that is involved in various signal transductions, including cell proliferation and cell invasion [178,179]. Various studies of gene expression and preclinical trials suggested that the MSL subtypes TNBC are more sensitive to Src inhibitors than any other TNBC subtypes [172]. The efficacy and safety of an Src inhibitor, dasatinib was investigated in a clinical trial (CA180059) in patients suffering from advanced TNBC. The phase II clinical trials showed a 9.2% clinical benefit rate with a 19% disease control rate [165,180]. Moreover, a smaller study using another Src inhibitor saracatinib was performed on TNBC patients but failed to deliver positive results [181]. 

#### 4.2.6. Immune Checkpoint Inhibitors

Immunotherapies have confirmed their efficiency in producing clinical responses, which were found evident from the treatment with immune checkpoint inhibitors. Treatment with immune checkpoint inhibitors helps in releasing the immune system from the inhibitory signals and rejuvenates the system against the tumors, as exhibited by various clinical studies employing monoclonal antibodies against CTLA-4, PD-1, and PD-L1 [182]. To date, many monoclonal antibodies were approved by FDA, which includes ipilimumab (anti-CTLA-4 antibody); pembrolizumab, nivolumab, and cemiplimab (anti-PD1 antibodies), and atezolizumab, avelumab, and durvalumab (anti-PD-L1 antibodies) [183,184]. In the current line-up, several studies reported that the combinatorial therapy of chemotherapeutics with the inhibitors of PD1/PD-L1 showed encouraging results in the treatment of early, locally advanced, and metastatic TNBC [182]. In this context, on the basis of IMPassion 130 trials, the FDA has approved the combination of an inhibitor of PD-L1 (Atezolizumab) and nab-paclitaxel for the treatment of TNBC. It was observed from the IMPassion130 trial (NCT02425891) that in the PD-L1+ population, the combination regimen exhibited a prolonged median OS of 25 months, a PFS of 7.5 months, and ORR of 58.9%, as compared to the single nab-paclitaxel group, which shows a median OS of 15.5 months, PFS of 5 months, and ORR of 42.6% [123,185]. Similarly, the I-SPY 2 trial (NCT01042379) was conducted involving a combination of pembrolizumab and paclitaxel vs. paclitaxel against early-stage TNBC. The study showed that a significantly higher pCR was exhibited by the combination group (71%) as compared to the control group (19%) [163]. The combination of an immune checkpoint inhibitor with an inhibitor of PARP offers great potential for uplifting the clinical benefit of TNBC patients. In a COLET (NCT02322814) clinical study, atezolizumab was combined with cobimetinib (MEK1/2 inhibitor) and nab-paclitaxel treating patients with locally advanced or metastatic TNBC. An interim analysis exhibited 34% ORR with the combination regimen as compared to only 29% ORR with nab-paclitaxel alone. In response to this study, other clinical trials are currently ongoing where the inhibitor of MEK (Binimetinib) is combined with pembrolizumab (NCT03106415) or avelumab (InCITe/NCT03971409) in patients with locally advanced or metastatic TNBC [182].

#### 4.2.7. Antiandrogens

As mentioned in Section 3.12, 20–40% of TNBC cases appeared positive for the androgen receptor (AR) [161]. According to Lehmann, such AR+ cancers are associated with the LAR subtype of TNBC. However, very limited studies were performed regarding the employment of AR inhibitors in targeting the LAR subtype of TNBC. A phase II trial, (NCT00468715) evaluating bicalutamide, an AR inhibitor, is currently ongoing in TNBC patients. If effective, this pathway could offer a potential nontoxic and targeted treatment approach against the LAR subtype of TNBC [172]. Another oral AR inhibitor, Enzalutamide, exhibited enhanced antitumor activity and better tolerance in metastatic AR+ TNBC. Traina et al., 2018, reported that enzalutamide showed 33% and 28% CBR at 16 and 24 weeks, respectively, along with 3.3 months of median PFS and 17.6 months of median OS [161]. Bonnefoi et al., 2016, reported that a combination of abiraterone acetate and prednisone showed a clinical benefit rate of 20% and a median PFS of 2.8 months in AR+ TNBC. Abiraterone acetate (AA) reduces the serum androgen levels by inhibiting 17-[α]-hydroxylase/17,20-lyase (CYP17) [186]. Other inhibitory AR targeting strategies against TNBC include several clinical trials evaluating orteronel, an inhibitor of nonsteroidal CYP17A1, which is essential for the synthesis of androgen. These trials involve monotherapy of orteronel for metastatic TNBC (NCT01990209) and a polytherapy of orteronel with enzalutamide for early-stage TNBC (NCT02750358). The same polytherapy is also being explored for the treatment of advanced TNBC (NCT01889238) [187]. Recent studies reported that the AR also interacted with numerous signaling pathways like PI3K/AKT/mTOR and MAPK pathways, and key proteins like BRCA1, BRCA2, FOXA1, and PTEN. Hence, a combination strategy could be evaluated for providing a synergistic approach against AR+ TNBC. In the MDA-MB-453 xenografts, a combinatorial therapy of rapamycin and enzalutamide were administered, which exhibited a potent antitumor efficacy [188]. Moreover, a Phase 1b/2 trial (NCT02457910) is ongoing, which comprises a combination of PI3K inhibitor (taselisib) and enzalutamide in AR+ metastatic TNBC patients. Similarly, the ENDEAR phase 3 trial (NCT02929576) was conducted to assess the efficacy of enzalutamide and paclitaxel combination in patients suffering from metastatic AR+ TNBC. Another phase 2b trial (NCT02689427) is ongoing, using the same combination profile but in patients suffering from early-stage AR+ TNBC [168].

From the above discussion, it came into focus that in both the preoperative as well as adjuvant settings, chemotherapy remained the cornerstone of treatment for TNBC patients. Also, trials are ongoing to determine the efficacy of distinct chemotherapeutic regimens either alone or in combination with newly identified targeted agents in both the neo-adjuvant as well as adjuvant settings. The chemotherapeutic strategies employed for the management of TNBC include inhibition of cell division and proliferation (taxanes), targeting DNA repair complex (anthracyclines, cyclophosphamide, and platinum compounds), and interference with DNA synthesis (antimetabolites), while the targeted therapy includes inhibitors of various targets like inhibitors of γ-secretase, Poly (ADP-ribose) polymerase-1, growth factors like EGFR, FGFR2, VEGF, PI3K/AKT/mTOR, Src kinase, immune checkpoints, androgen receptor, etc. It is also assumed that results from such ongoing trials will significantly provide a platform for the management of TNBC in a high-risk population. 

We have summarized different classes of anticancer drugs (chemotherapeutic and targeted therapeutics) and reflected their suitability for different subtypes of TNBC [133] in Table 1, and in Table 2, we have showcased various clinical trials that were employed against TNBC using a recent combination regimen of chemotherapeutics and targeted therapeutics.

## 5. Nanotechnology: Cavalry for TNBC Therapy 

Conventional therapies deal with various shortcomings that include rapid drug degradation and simultaneous elimination from the system, poor bioavailability due to non-specific targeting and biodistribution, systemic toxicity, and the emergence of drug resistance. In addition to these, cancer cells also show resistance towards chemotherapy because of overexpression of efflux pumps, intensified drug biotransformation, and modification of target sites [212,213]. Owing to these shortcomings with the conventional delivery system, several nanotechnology-based approaches have emerged for the efficient treatment of TNBC. 

**Figure 5:**
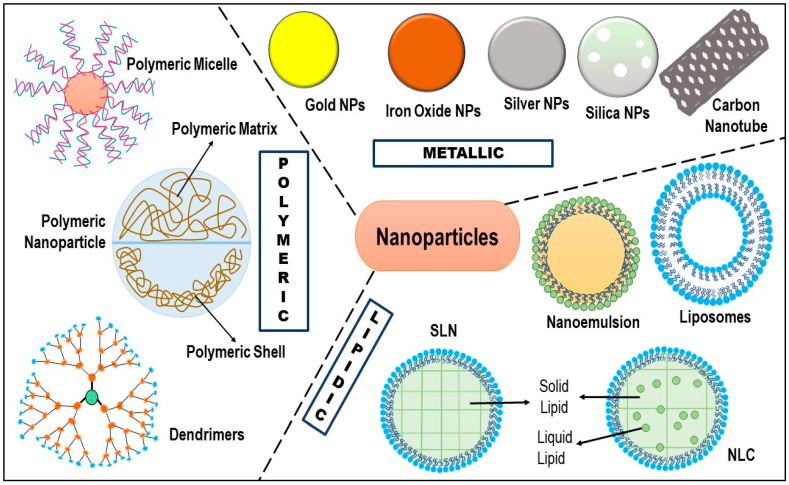
Different types of nanoparticles administered for the effective treatment of triple-negative breast cancer (TNBC) [212].

Nanoparticles are carriers with dimensions ranging from 1 to 1000 nm [214]. They are equipped with exclusive attributes that include enhanced bioavailability, improved internalization into the tumor site, site-specific delivery, anti-metastatic activity, and evading multi-drug resistance (MDR) [212,215,216]. The various nanotechnology-based drug delivery system developed for the treatment of TNBC includes liposomes, dendrimers, polymeric micelles, polymeric nanoparticles, carbon nanotubes, metallic nanoparticles, nanoemulsions, solid lipid nanoparticles (SLN), and nanostructured lipid carriers (NLC) (Figure 5).

Widely used antineoplastic drugs for the treatment of TNBC include paclitaxel, docetaxel, cisplatin, carboplatin, bortezomib, doxorubicin, etc., but despite their therapeutic efficacy against TNBC, they do suffer limited usage due to poor bioavailability. Nanoparticles can improve bioavailability, thereby increasing their therapeutic utilization [217]. Their small size provides a large surface area that enhances their permeation through the gastrointestinal membrane and increases the dissolution of the drug. Moreover, the presence of lipids in the lipidic nanoparticles stimulates the contraction of the gall bladder and releases biliary and pancreatic secretion that results in the formation of crude emulsion that aid in drug solubilization. Further, the lipids follow lymphatic transport and bypass the hepatic first-pass metabolism, which also aids in increased bioavailability [218]. Secondly, the presence of various excipients in nanoparticles such as Tweens, Spans, Poloxamers, TPGS 1000, etc., inhibits P-gp efflux pumps, and, as a result, limits the elimination of the drug from the body and increases the drug concentration at the target site, thereby improving the drug-bioavailability. Also, excipients like Solutol HS 15 and TGPS 1000 have the ability to inhibit the cytochrome P450 based pre-systematic metabolism within the enterocytes, which also leads to an increase in drug bioavailability [212]. 

**Figure 6:**
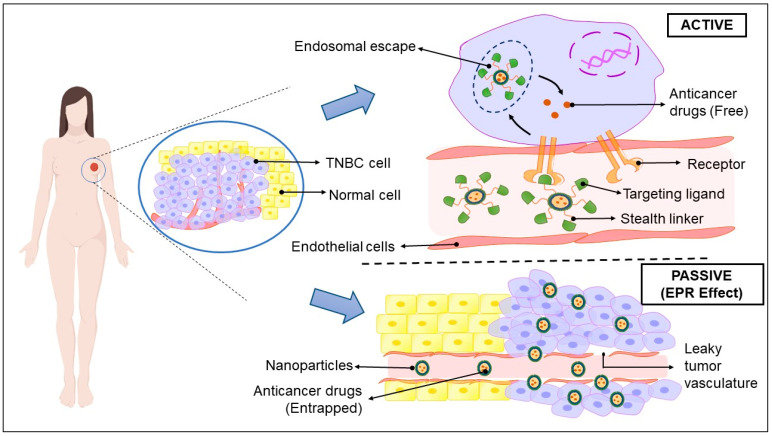
Schematic illustration of the passive targeting (EPR effect) and the active targeting (receptor-ligand effect) into the TNBC cells.

The basic mechanism of any anticancer drug is to restrict the proliferation of rapidly multiplying tumor tissues or cells. However, in the due course, they also inhibit the other proliferative cells of the body, which includes bone marrow cells, hair follicles, etc., resulting in systemic side effects such as low platelet count, alopecia, and anemia. The nanoparticles can be employed to prevent the systemic side effects and emphasize targeted delivery of the drug at the tumor site [219]. Nanoparticles target the tumor site by active targeting and passive targeting (Figure 6). The nanoparticles are passively targeted via the EPR effect, which enables them to accumulate within the tumor vasculature (100–600 nm) [220]. At the same time, the nanoparticles are actively targeted to the overexpressed receptors on the cancer cells via surface functionalization with specific ligands, thus increasing their cellular uptake, facilitating a site-specific drug delivery inside the tumor cell, and avoiding off-target toxicities [212,221,222]. 

In recent times, gene delivery has appeared therapeutically helpful in treating TNBC by inducing oncogene suppression and tumor suppressor gene promotion [223]. In this context, small interfering RNA (siRNA) therapy exhibited an approachable result in the suppression of certain oncoproteins. However, its clinical application is hindered by rapid degradation, low cellular uptake, and insufficient endosomal escape [224]. Nanoparticles *viz.* liposomes, dendrimers, and SLNs in this respect provide a shield around the labile genetic material that prevents their degradation and results in enhanced cellular uptake [225,226]. One term that defines TNBC more accurately than any other type of breast cancer is metastasis more specifically in the lung, brain, and bones. It is observed from various reports that approximately 36% of TNBC cases show metastatic central nervous cell lesions [6,227]. Brain metastasis dictates a catastrophic reverberation of TNBC, reducing the chances of patient survival. The innate disability of systemic chemotherapeutics to bypass the blood-brain barrier (BBB) makes it difficult to treat brain metastasis of TNBC [228]. Moreover, the existence of overexpressed P-gp efflux pump or breast cancer resistance protein at BBB also limits the entry of chemotherapeutics into the brain [229]. In this context, extensive works are going on for the development of targeted nanoparticles owing to their property of tunable surfaces. Such nanoparticles are functionalized with suitable ligands for mediating a receptor-mediated uptake at BBB via glutathione (GSH) receptors, insulin receptor, and low-density lipoprotein (LDL receptor, and transferrin receptor) [230]. 

It could be inferred from this section that nanotechnology provides a potential strategy to overcome various drawbacks specific to conventional therapies that include lack of tumor targetability and systemic side effects. Clinically, several approved nanomedicines are used in health institutes worldwide, like liposomal doxorubicin (Doxil^TM^) [231], Nab-paclitaxel (Abraxane^TM^) [232], and polyethylene glycol-1 Asparaginase (Oncaspar^TM^) [164]. Hence, it was revealed that nanoparticles have exhibited their clinical potential in the field of diagnostics and therapy, which eventually led to their marketing. It was observed that the value of the nanomedicines was found to be USD 53 billion in the year 2009, which was then estimated to be USD 334 billion by 2025. It was further observed that the first–generation nanomedicines had started going off-patent, which initiated the entry of the new generation nanomedicine in the healthcare market, and which will eventually create more market value. In accordance with Grand View Research Report, USA remained the leader of the nanomedicine industry with 46% of total international nanomedicine revenue followed by Europe and Japan. Moreover, it was observed that anticancer nanomedicines still remained the largest niche of the nanomedicine market with almost 50% revenue, which will continue to grow in the coming years [233]. Although the nanomedicine market is blooming, the global spending on conventional medicines is still higher, with an estimation of USD 1.4 trillion by 2020, where the USA alone spends approximately USD 350 billion. However, like the nanomedicines, the spending on conventional drugs was found to be higher in the field of cancer, which is approximately USD 150 billion [234]. Hence, we could estimate that in the future, if the spending on nanomedicine keeps rising at the same pace, it could contribute significantly to the field of medicine with affordable care.

### 5.1. Liposomes

**Figure 7:**
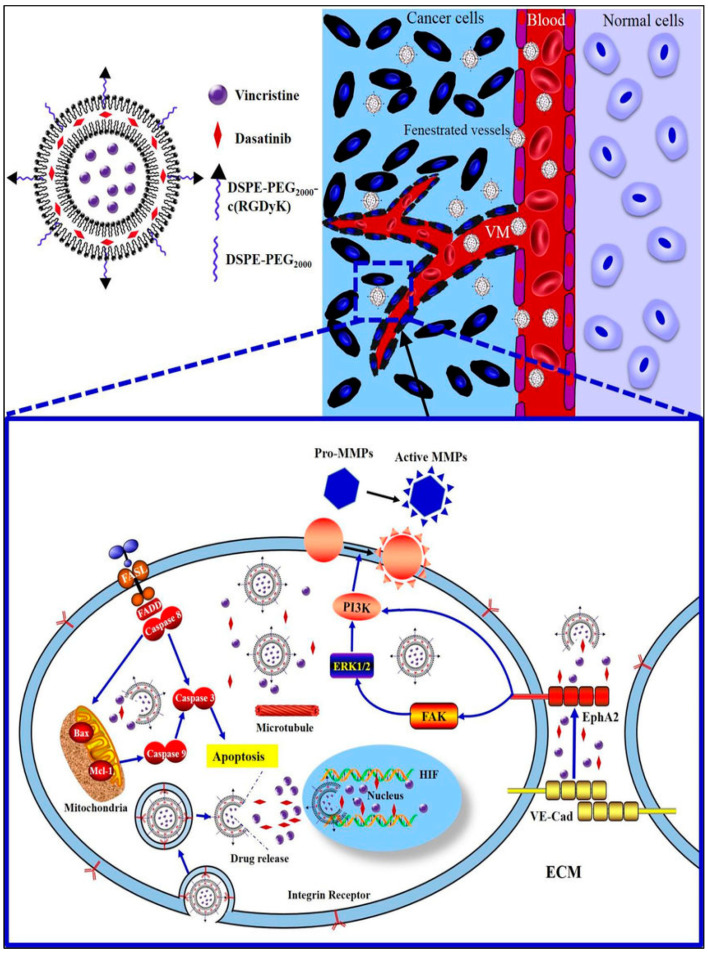
Schematic diagram showing mechanism of functional vincristine plus dasatinib liposomes in the elimination of VM channel for the treatment of TNBC. This work is licensed under Creative Commons Attribution 3.0 License (CC BY 3.0). This work is attributed to Ref. [237].

Liposomes are spherical lipid-based nanocarriers comprised of an inner aqueous core and outer phospholipid bilayer with a diameter ranging from 50 to 100 nm [235]. They can encapsulate both hydrophilic and hydrophobic drugs, genes, etc. The liposomes also exhibit certain advantages as compared to conventional dosage forms, which include increased circulation time, biocompatibility, escape from the reticuloendothelial system, and decreased systemic toxicity [236]. However, liposomes do exhibit certain demerits such as low drug loading capacity due to the presence of little space within the membrane, poor stability, poor reproducibility, difficulty in sterilization, and the possibility of oxidation of phospholipids [220]. Zheng et al., 2015, developed a liposome loaded with a combination of dasatinib (Src inhibitor), and vincristine (mitotic inhibitor) that exhibited targeted elimination of vasculogenic mimicry (VM) channels, resulting in prevention of relapse of TNBC by inhibiting VM indicators including vascular endothelial-cadherin (VE-Cad), FAK, PI3K, MMP-2, and MMP-9 (Figure 7). In addition, the liposomes demonstrated a delayed-release profile that enabled maximum drug delivery at the tumor site, facilitating maximum apoptosis with minimum leakage in circulation. Hence, drug-loaded liposomes can act as a potential therapeutic platform against TNBC [237]. 

Parvani et al., 2015, fabricated F3 peptide targeted liposomes encapsulated with β3 integrin siRNA that silenced the expression of β3 integrin, which was found overexpressed in TNBC as well as reduced in TGF-β mediated EMT. Moreover, tumor cells treated with targeted liposomes showed no sign of metastasis and relapse even after 4 weeks post-treatment, as compared to untreated cells. Hence, targeted liposomes serve as a promising therapeutic strategy to combat TNBC [238]. Doddapaneni et al., 2015, formulated cationic PEGylated liposomes loaded with gambogic acid (GA) for intravenous delivery to overcome its problem of poor aqueous solubility for the effective treatment of TNBC. The liposomes showed >50% reduction of tumor volume and a 1.7-fold decrease in tumor weight when compared with GA alone. Hence, it was concluded that GA-loaded cationic liposomes provide a significant effect against TNBC when administered intravenously [239]. Mohammad and his associates, formulated in 2019 irinotecan-loaded liposomes that bypassed the blood -tumor-barrier, improved the local exposure of irinotecan, and prevented brain metastasis of TNBC. The irinotecan-loaded liposomes exhibited enhanced plasma drug exposure with 17.7 ± 3.8 h mean residence time (MRT), as compared to free irinotecan, which is only 3.67 ± 1.2 h. Further analysis showed increased accumulation of irinotecan-loaded liposomes in the metastatic lesion, as compared to free irinotecan. Hence it was inferred that liposomes delivered a sustained release profile by developing a depot within the tumor region, which later gets accumulated in brain metastasis, and which delays TNBC progression [227]. Yan et al., 2019, fabricated DSPE-PEG2000-tLyp-1 peptide-functionalized liposomes encapsulated with miRNA that can silence the Slug gene, for the treatment of TNBC. The spherical nanosized liposomes (120 nm) were effectively captured by the TNBC cells and were targeted to mitochondria where the miRNA silenced the expression of the slug gene and resulted in the inhibition of the TGF-β1/Smad pathway and invasiveness, and hence acted as an effective approach for the treatment of TNBC [240]. Burande et al., 2020, prepared paclitaxel and piperine co-loaded liposomes for improving the bioavailability of paclitaxel, which was further coated with TPGS for targeting EGFR, which is overexpressed in TNBC. The percent encapsulation of paclitaxel and piperine in the liposomes was found to be 31% and 73%, respectively. The targeted liposome showed increased cellular uptake and improved cytotoxicity profile in comparison to the non-targeted counterparts. In addition, the IC_50_ of targeted liposomes was found to be 9.309 ± 0.85 μg/mL, as compared to paclitaxel alone (IC_50_ = 54.150 ± 1.320 μg/mL). Hence, the liposomes co-loaded with paclitaxel-piperine provide a potential platform for the successful loading of various combinations of drugs for the effective treatment of TNBC [241]. 

### 5.2. Dendrimers

Dendrimers are highly branched symmetrical nanostructures ranging from 10 to 100 nm in diameter. Like liposomes, they are also composed of the hydrophobic core and hydrophilic periphery [242], making them suitable for entrapping both hydrophilic and hydrophobic drugs along with siRNA, genes, vaccines, etc. [236]. Jain et al., 2019, developed phosphorus, and polyamidoamine dendrimer loaded with PLK1 siRNA, for targeting polo-like kinase 1 (PLK-1), a potential target for TNBC. The dendrimers showed enhanced internalization in tumor cells due to their cationic nature, which favors their interaction with the tumor cell, as compared to the siPLK1 solution. Hence, it was concluded that dendrimers serve as a potential carrier for the distribution of siRNA in the TNBC cells [243]. Liu et al., 2019, developed poly(amidoamine) dendrimer encapsulated with doxorubicin for the treatment of TNBC. Due to the cationic nature, the dendrimers showed increased internalization in the tumor cells, and the attachment of EGFR-binding peptide 1 and trans-activating transcriptional activator to the dendrimer further enhanced their cellular uptake, which resulted in increased accumulation of drug at the cancer site in vivo, along with effective inhibition of cancer growth and prolonged survival. Hence, it was concluded that dendrimers can be modified or functionalized with suitable ligands for the effective targeting of drugs for the treatment of TNBC [244]. Torres-Perez and his associates, prepared in 2020 PAMAM dendrimers loaded with methotrexate and D-glucose for the treatment of TNBC. The spherical nano-ranged dendrimers (∼30 nm) were taken up by the tumor cells through the EPR effect and showed 2-fold increased tumor cell internalization because of the presence of surface positive charge (13 to 19 mV), as well as glucose moiety. In addition, the dendrimers showed 20% less cell viability as compared to free methotrexate. Hence, dendrimers provide an effective platform for targeted therapy against TNBC [245]. Despite showing great potential as a drug delivery system for cancer treatment, dendrimers demonstrate cytotoxic as well as hemolytic characteristics. In addition to these, dendrimers also show less yield. However, it was observed that such drawbacks are bypassed by surface modifications [246].

### 5.3. Polymeric Micelles

Polymeric micelles are nanometric amphiphilic colloidal nanocarriers composed of an interior hydrophobic core and an exterior hydrophilic surface, which undergo self-aggregation above CMC [247]. In diameter, the micelles range from 10 to 100 nm, which enables them to get accumulated in the tumor site via extravasation. Also, like previously stated nanocarriers, polymeric micelles can entrap both lipophilic and lipophobic drugs with enhanced loading capacity and retention efficacy in the systemic circulation [248]. Despite such merits, polymeric micelles exhibits similar drawback as liposomes, such as low drug loading capacity and poor stability, which might lead to rapid release of the encapsulated drug in vivo [249,250].

Kutty et al., 2015, prepared suberoylanilide hydroxamic acid (SAHA) and paclitaxel co-loaded hybrid micelle system for providing a synergistic effect against mesenchyme-like TNBC. The polymeric micelles showed a burst release profile, representing a rapid release of drugs from the micelle, indicating a rapid onset of action. It was observed that approximately 50–60% of drugs were released at 24 h from the hybrid micelle, as compared to the non-micellar combination. Moreover, a synergistic effect was observed from hybrid micelle (IC_50_ = 0.52 μg/mL), as compared to non-micellar combination (IC_50_ = 3.071 μg/mL). Hence, polymeric micelles act as an encouraging therapeutic platform against TNBC [251]. Su et al., 2016, fabricated doxorubicin and docetaxel co-loaded polymeric micelle comprising of polyethylene glycol, poly (D, L-lactide-co-glycolide), and porphyrin. The drug-loaded polymeric micelles showed an increased internalization and accumulation in the tumor site through EPR and offered an effective drug release profile in acidic organelles. In addition, a synergistic anti-tumor activity was observed from the polymeric micelles, as compared to free drugs. Hence porphyrin-based polymeric micelle co-loaded with dual drugs provide a promising platform for tackling the stubborn TNBC [252]. Paulmurugan et al., 2016, fabricated 2-hydroxyethyl acrylate (HEA) and 2-ethylhexylacrylate (EHA) copolymerized orlistat loaded micelle to improve its aqueous solubility and enhance its therapeutic efficacy against TNBC. The polymeric micelle was further functionalized with folic acid for improved targeting. MDA-MB-231 cell line studies showed that folate-linked polymeric micelle exhibited increased apoptosis and decreased tumor volume, as compared to free orlistat, which indicated improved delivery and enhanced bioavailability of orlistat from the NPs. It was inferred that orlistat-loaded polymeric micelle acted as an effective drug carrier in TNBC therapy [253]. Brinkman et al., 2016, prepared aminoflavone (AF)-loaded EGFR targeted polymeric micelle to minimize the pulmonary toxicity associated with aminoflavone for effective treatment of TNBC. The small-sized polymeric unimolecular micelle showed effective cellular uptake by endocytosis in presence of endosomal pH as compared to blood pH, concomitant with increased stability. The AF-loaded micelle was further functionalized by GE11, which resulted in increased targetability and apoptosis as compared to free AF. Hence, AF-loaded EGFR targeted micelle served as an effective platform for the EGFR-overexpressed TNBC [254]. Martey et al., 2017, formulated curcumin derivative RL71 loaded styrene-maleic acid (SMA)-based micelles for the treatment of TNBC, which exhibited enhanced biodistribution and a 16-fold increased accumulation of the drug in the cancer site in comparison to free RL71 because of the nanometric size of the micelle that facilitated enhanced accumulation via extravasation across the large fenestration of the tumor. In addition, the SMA-RL71-micelle exhibited increased apoptosis with no cytotoxicity [255]. Godugu et al., 2017, fabricated a honokiol-loaded nanomicellar formulation to improve its bioavailability for the effective treatment of TNBC. It was observed that the honokiol-loaded nanomicelles exhibited increased absorption, which resulted in increased oral bioavailability (Cmax = 4.06 fold; AUC = 6.26), in comparison to the free drug (40 mg/kg). In addition, the nanomicelles also showed a distinct reduction in tumor volume and weight in comparison to free honokiol. Hence, the nanomicelles provide an effective approach for increasing the oral bioavailability in the treatment of TNBC [256]. Chida et al., 2018, formulated epirubicin (EPI)-loaded polymeric micelles functionalized with pH triggered moiety to inhibit the axillary lymph node metastasis in TNBC. The polymeric micelles underwent selective accumulation and penetration in primary tumors and vascularized axillary lymph node metastasis. The pH-triggered moiety further facilitated drug release in the acidic tumor microenvironment, sparing the healthy tissues [257]. Zuo et al., 2021, developed halofuginone hydrobromide (HF) loaded TGPS polymeric micelles to improve its aqueous solubility and enhance its therapeutic efficacy against TNBC. Polymeric micelles showed a sustained drug release profile with excellent stability and biocompatibility in vivo. They also exhibited enhanced tumor growth inhibition (68.17%) in comparison to free drugs. Hence it was concluded that polymeric micelles hold significant clinical potential for the treatment of TNBC [258]. 

### 5.4. Polymeric Nanoparticles

**Figure 8:**
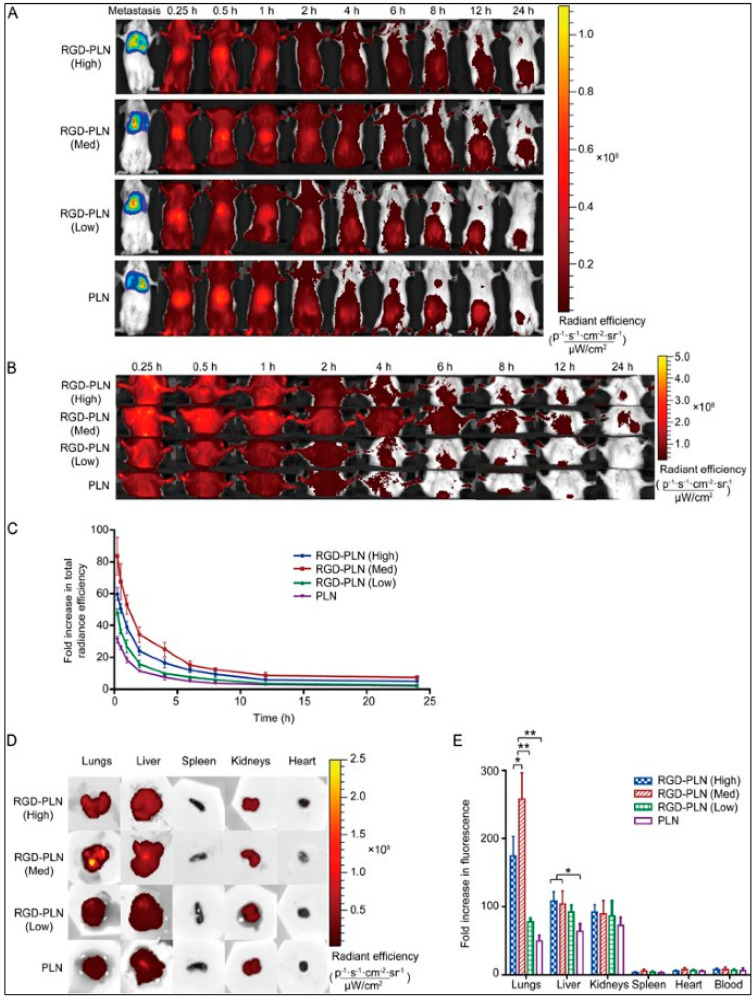
Fluorescent imaging of ICG labeled DOX-MMC-NP biodistribution in the MDA-MB 231-luc-D3H2LN lung metastasis SCID model of TNBC. (**A**) Biodistribution images of the whole body upto 24 h through Xenogen IVIS Spectrum System 100 with Ex: 745 nm and Em: 820 nm. (**B**) Magnified images of NPs accumulation in the lungs for 24 h. (**C**) A quantitative representation of DOX-MMC-NP biodistribution in lung metastatic region up to 24 h. (**D**) Qualitative representation of DOX-MMC-NP biodistribution in various organs ex vivo for 4 h. (**E**) Quantitative representation of DOX-MMC-NP biodistribution in various organs ex vivo for 4 h (* *p* < 0.05, ** *p* < 0.01). This work is licensed under the Creative Commons Attribution-Non-Commercial-No Derivative Works 3.0 Unported License. http://creativecommons.org/licenses/by-nc-nd/3.0/ (accessed on 14 June 2021). This work is attributed to ref. [270].

Polymeric nanoparticles are solid colloidal nanocarriers whose diameter ranges from 50 nm to 10 μm. The drug is either adsorbed or dissolved or entrapped or encapsulated within the polymeric matrix [259]. Polymeric nanoparticles provide numerous advantages against conventional dosage forms, such as increased drug permeability, enhanced targetability, controlled drug delivery, and reduced systemic cytotoxicity [260]. In addition to this, polymeric nanoparticles show some drawbacks which include aggregation of toxic monomers, their toxic degradation, and the presence of residual material [261]. Hence, to minimize such drawbacks, many biocompatible polymers are used for the fabrication of polymeric nanoparticles to acquire rapid and effective clinical translation with less toxicity such as chitosan [262,263], alginate [264], pectin, gelatin [265], polyethylene glycol (PEG) [266], poly-lactic acid (PLA), and poly-lactic-co-glycolic acid (PLGA) [267]. Gupta et al., 2017, formulated chitosan nanoparticles loaded with paclitaxel (PTX-CS-NP) to provide a better and secured delivery system for the treatment of TNBC, as chemotherapeutics tend to exhibit systemic toxicity. The hemolytic toxicity profile of PTX-CS-NP was found to be 4-fold less than the free PTX, which showed that the NP is biocompatible and safe for delivery. Also, the PTX-CS-NP showed a sustained drug release profile where approximately 60% of the drug was released within 24 h, which hence indicated that CS-NP followed a controlled drug delivery. Additionally, the IC_50_ of PTX-CS-NP and free PTX were found to be 9.36 ± 1.13 μM, and 14.755 ± 1.68, respectively. It was thus concluded that biodegradable polymeric nanoparticles provide a robust and safe platform for the treatment of TNBC [268]. Zhou et al., 2017, developed a PLA-b-PEG nanoparticle loaded with erlotinib (Ei), and DOPA- doxorubicin (DOPA-Dox) to provide a sequential delivery for the effective treatment of TNBC as well as to reduce their systemic toxicity. The drug release profile showed that approximately 80% of erlotinib was released within 4 h of administration, whereas only 20% of doxorubicin was released up to 24 h of administration of polymeric nanoparticles. Further, the biodistribution study exhibited that the NPs initiate accumulation inside the tumor within 1 h of administration, which continued up to 24 h due to the EPR, however, the NPs accumulated to other organs get cleared out after 15 days of post-administration. It was concluded that polymeric nanoparticles can entrap combination drugs with sequential delivery for the safe and controlled therapy of TNBC [269]. Zhang et al., 2017, prepared polymer-lipid hybrid nanoparticles co-loaded with doxorubicin (DOX) and mitomycin C (MMC) and surface conjugated with Arg-Gly-Asp peptide (RGD) for preventing lung metastasis of TNBC. In comparison to free drugs, the NPs exhibited a 31-fold decrement in lung metastases as observed through bioluminescence imaging, concomitant with a 57% longer median survival time (Figure 8). Hence, NPs showed more accumulation in lung metastases due to their ability to target both tumor vasculature and tumor cells. Thus, it was concluded that dual-targeted RGD polymeric nanoparticles significantly prevented the lung metastasis of TNBC and prolonged the survival of the host [270]. 

Rad et al., 2020, prepared mPEG-PLGA co-polymers-based piperine-loaded nanoparticles for increasing the solubility of piperine and effective treatment of TNBC. The smaller size of the polymeric nanoparticles (32–82 nm) allowed them to undergo passive diffusion into the tumor site while bypassing its toxic retention in organs like the kidney, liver, and spleen. Additionally, the piperine-loaded polymeric NPs inhibited the growth of TNBC and induced apoptosis while sparing normal fibroblast. Hence, polymeric nanoparticles provide a platform for delivering a cytotoxic drug in the tumor site with increased solubility and enhanced bioavailability [271]. Zhou et al., 2020, developed PLGA-TPGS NPs loaded with quercetin for effective treatment of TNBC via oral administration. The in-vitro study revealed that the quercetin-loaded polymeric NPs exhibited a sustained drug release profile, as compared to free quercetin, thereby allowing proper utilization of the quercetin by NPs and improving its medical value. Also, quercetin-loaded PLGA-TPGS NPs showed enhanced inhibition of tumor growth as supported by an increased inhibition ratio of tumor (67.88%), along with few lung metastasis colonies. Moreover, quercetin-loaded PLGA-TPGS NPs provided an inhibitory effect upon the migration of uPA (Urokinase-type plasminogen activator) knockdown on TNBC cells, hence, providing an enhanced anticancer and significant antimetastatic activity for the treatment of TNBC [272].

### 5.5. Carbon Nanotubes

Carbon nanotubes (CNTs) were identified as a tool in biomedical application in 1991 by Iijima, a Japanese scientist. Carbon nanotubes are comprised of carbon atoms that are arranged in a series of benzene rings, which are further rolled up into a tubular-like structure. It was found that the carbon nanotubes belong to the fullerenes family. Carbon nanotubes are further divided into single-walled carbon nanotubes (SWCNTs) and multi-walled carbon nanotubes (MWCNTs) based on the number of carbon layers [273]. In recent times, CNTs are widely used as the delivery system of drugs, nucleic acids, proteins, peptides, etc. In addition, they can be easily modified by surface functionalization in order to provide tunable characteristics like low toxicity and non-immunogenicity [274]. It was further observed that besides enhancing the therapeutic activity of carbon nanotubes, the surface functionalization also increases their aqueous solubility, biocompatibility, and biodegradability, which were considered the drawback of CNTs [275]. 

Fahrenholtz et al., 2016, developed platinum-acridines (PA) loaded carbon nanotubes (PA-CNTs) to treat TNBC effectively. It was observed that the CNTs showed increased drug loading capacity and enhanced accumulation in the tumor site. Also, the CNTs exhibited increased cytotoxicity and apoptosis in the TNBC cells (MDA-MB-231, MDA-MB-468, SUM159, BT20), as compared to free PA [276]. Badea et al., 2018, fabricated cisplatin (CDDP) loaded multiwalled carbon nanotubes (MWCNTs) to overcome TNBC cells resistance. It was observed that the MWCNT-COOH-CDDP showed decreased cellular viability after 48 h as compared to free CDDP. Also, the MWCNT-COOH-CDDP showed decreased expression of caspase-3 and p53, followed by down-regulation of NF-κB in cells, promoting apoptosis [274]. Singhai et al., 2020, prepared Hyaluronic acid (HA) and α-Tocopheryl succinate (α-TOS) coated doxorubicin-loaded multi-walled CNTs to treat CD44 receptor overexpressed TNBC. It was observed that the functionalized doxorubicin-loaded CNTs exhibited increased cellular uptake, enhanced apoptosis (Annexin V/PI assay; 52.69 ± 4.86%; *p* < 0.005), and significant growth inhibition effect (SRB assay; GI50; 0.810 ± 0.017; *p* < 0.001), as compared to non-functionalized doxorubicin-loaded CNTs (Annexin V/PI assay: 15.34 ± 3.23%; SRB assay; GI50; 1.965 ± 0.042) and free doxorubicin (Annexin V/PI assay: 06.13 ± 1.4%; SRB assay; GI50: 2.621 ± 0.153) [277]. Luo et al., 2021, developed ginsenoside (Rg3) loaded CNTs for targeting PD-1/PD-L1 signaling pathways for the effective treatment of TNBC. It was observed that the Rg3-CNTs exhibited decreased TNBC cell viability, reduced colony formation, and increased apoptosis as compared to free Rg3. Further, it was observed that Rg3 impaired the upregulation of PD-L1 in activated T-cells. Moreover, treatment with CNTs reduced the levels of interleukins (IL-2, Il-9, Il-10, IL-22, and IL-23) and interferons (IFN-ϒ), thereby attenuating the PD-L1 signaling pathway [278].

### 5.6. Metallic Nanoparticles

Metallic nanoparticles serve as an emerging platform in diagnostic and therapeutic assay because of their electrical, magnetic, optical, and thermal properties. Various metallic NPs devices have distinct molecular mechanisms involving the generation of intracellular reactive oxygen species (ROS), an increase of oxidative stress, and inducing apoptosis in tumor cells. The metallic NPs can also undergo surface modification via conjugation to expands their utility in clinical applications [279]. In addition, metallic NPs tend to induce hyperthermia, thereby increasing the temperature of the tumor cells and destroying them, thus inhibiting cell growth. However, some metallic NPs possess intrinsic anti-cancer activity. Various metallic nanoparticles were employed for the treatment of TNBC, like gold (Au), silver (Ag) [280], iron (Fe_2_O_3_), zinc (ZnO), titanium dioxide (TiO2), etc. [242]. Sarkar et al., 2017, prepared gold nanoparticles (AuNM) functionalized with an inhibitor of tyrosine kinase, ZD6474, for treating TNBC. It was observed that at pH 5.2, AuNM exhibited a slow and sustained release of ZD6474 (82% following 45 h), facilitating the proper utilization of the drug and its delivery in the acidic tumor microenvironment. Also, AuMN showed targeting due to their low cytotoxicity and immunogenicity. In addition, the fluorescence signals showed enhanced retention of AuMN as compared to the control. The ZD6474 functionalized AuMN further inhibited tumor growth and prevented metastasis without causing any haemotoxicity [281]. Laha et al., 2019, prepared a curcumin-loaded metal-organic framework (NMOF-3) tagged by folic acid (IRMOF-3@CCM@FA) for improving the solubility of curcumin and treatment of TNBC. IRMOF-3@CCM@FA showed a suitable drug loading content of 52% because of their extensive large surface area along with the presence of -NH_2_ over their surface. Furthermore, IRMOF-3@CCM@FA showed 55% and 31% drug release in acidic pH and physiological pH, respectively, indicating an enhanced intracellular accumulation of curcumin within the acidic tumor microenvironment. Also, the IRMOF-3@CCM@FA showed enhance apoptosis and targeted delivery of curcumin, as compared to free curcumin and non-targeted curcumin delivery [282]. Swanner et al., 2019, prepared silver nanoparticles (AgNPs) to selectively treat the TNBC without affecting the normal breast epithelial cells. AgNPs exhibited accumulation to both TNBC and healthy breast cells, but facilitate rapid degradation only in the cells of TNBC. Moreover, internalization of AgNPs within the TNBC showed depletion of cellular antioxidants, causing endoplasmic reticulum stress and facilitating apoptosis, however, such damage was not observed in the non-malignant breast cells. Hence AgNPs provided a promising curative platform for treating TNBC [283]. However, it was further observed that there are certain drawbacks associated with metallic nanoparticles that can compromise their therapeutic activity for cancer treatment. Such drawbacks includes poor stability as they are thermodynamically unstable systems, and chances of accumulating impurity during their synthesis, because the metallic nanoparticles are highly reactive and can cause irritation. Also, it becomes challenging to synthesize the nanoparticles in an encapsulated form [284].

### 5.7. Nanoemulsion/Self-Nanoemulsifying Drug Delivery System (SNEDDS)

**Figure 9:**
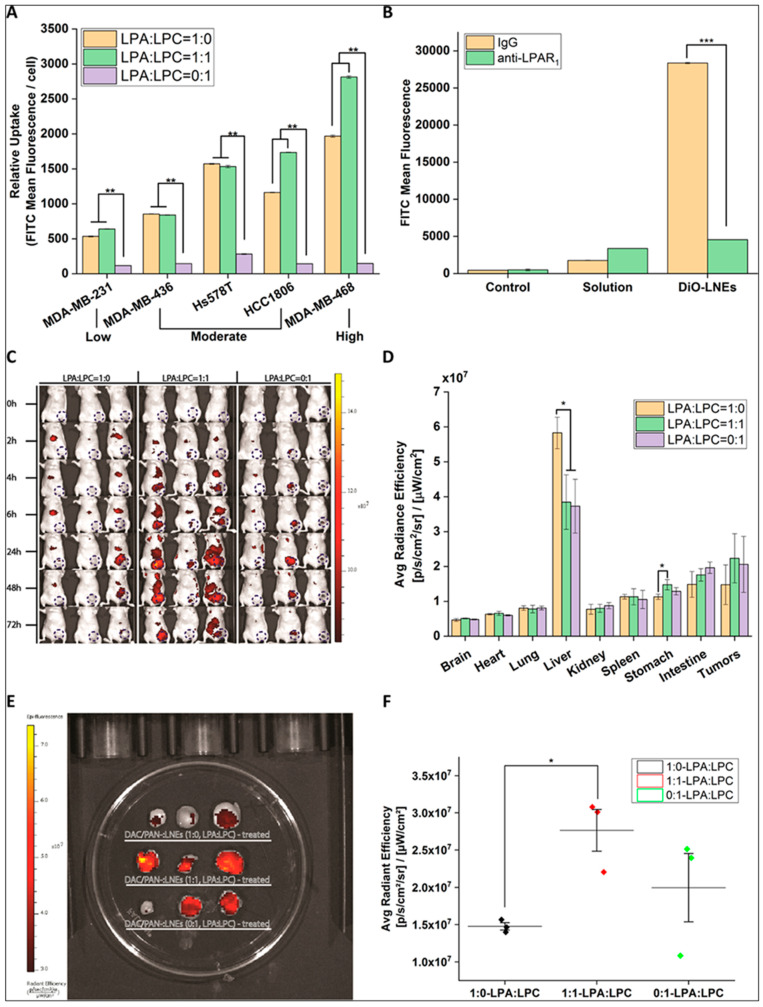
Cellular uptake and biodistribution of lipid nanoemulsion (LNE). (**A**) Different levels of LPAR1 by flow cytometry in TNBC cell lines after incubated for 2 h with Rho-LNEs. Similarly, (**B**) shows cellular uptake of LNEs via flow cytometry, which was pre-incubated with either IgG or anti-LPAR1 at 10 μg/mL concentration for about 1 h followed by incubation with DiO-LNEs. (**C**) Whole body DiR-LNEs radiance for 72 h post IV injection. (**D**) Biodistribution of DiR-LNEs in various organs, which were later quantified ex vivo by IVIS for 72 h post IV injection. (**E**,**F**) Radiance from DiR-LNEs treated tumors. (* *p* < 0.05, ** *p* < 0.01, *** *p* < 0.001). Reprinted (adapted) with permission from Ref. [294] Copyright 2019 American Chemical Society.

Nanoemulsions are lipid-based systems comprised of dispersion of two immiscible liquids that are thermodynamically stabilized by emulsifying agents with a diameter in the range between 100 and 500 nm [285]. Another form of nanoemulsions that are used in cancer therapy is the self-emulsifying drug delivery system (SEDDS). They are considered as an isotropic blend of oil, surfactant, and co-surfactants that form an oil-in-water nanoemulsion when exposed in an aqueous phase under mild agitation [286]. They range within a diameter of 10–200 nm [287]. The nanoemulsion offers therapeutic advantages over conventional dosage form, which includes small size, increased aqueous solubility, good physical stability, increased bioavailability, reduced cytotoxicity, and overcoming the multi-drug resistance (MDR) [285,288]. They can further be modified or functionalized with ligands to target the tumor cells [289]. Despite the advantages, nanoemulsion and SNEDDS experience certain limitations. The presence of surfactant or mixture of surfactants in the nanoemulsion and SNEDDS may lead to irritation in GIT, resulting in toxicity. Also, the presence of a high concentration of surfactant may lead to degradation of the therapeutics, resulting in instability. Such problems could be solved by optimizing the delivery system with a smaller amount of GRAS-regulated surfactants [290,291]. Pereira et al., 2018, developed an omega 3-fatty acid derivative loaded nanoemulsion against TNBC. The nanoemulsion showed 99.9 ± 2.3% entrapment efficiency and indicated a complete drug encapsulation into dispersed oil phase along with 150 nm particle size, facilitating enhanced tumor cell accumulation of lipophilic omega 3 fatty acid derivative via oral administration. Moreover, the nanoemulsion showed a 50% reduced tumor weight as compared to the free derivative of omega 3 fatty acids [292]. Timur et al., 2019, formulated doxorubicin hydrochloride and LyP-1 co-loaded self-micro emulsifying drug delivery system (SMEDDS) to mediate active targeting of TNBC cells. The particle size of 100 nm mediated lymphatic uptake of the SMEDDS, thereby bypassing the first-pass metabolism and offering enhanced bioavailability. Moreover, the SMEDDS showed enhanced in vivo cytotoxicity in p32 expressing TNBC cells. Also, in comparison to free drugs, SMEDDS showed reduced tumor growth and metastasis. Hence, SMEDDS could be served as a therapeutic platform for combination drug delivery via the lymphatic pathway for the treatment of TNBC [293]. Kim et al., 2019, fabricated decitabine (DAC) and panobinostat (PAN) co-loaded lipid nanoemulsion (LNEs) for the treatment of TNBC. The LNEs showed increased stability, enhanced internalization in tumor cells, without affective liver, and spleen, and controlled release delivery system of both the drugs, thus facilitating proper utilization. Moreover, the LNEs showed increased inhibition of tumor growth of M subtype TNBC via restoration of CDH1/E-cadherin, and suppression of forkhead box M1 (FOXM1) expression (Figure 9) [294]. 

Saraiva et al., 2021, prepared edelfosine nanoemulsions (ET-NEs) for the treatment of TNBC. The nanoemulsion of particle size of 120 nm and neutral zeta potential exhibited an enhanced passive targeting into the tumor site via the EPR effect, with fewer chances of opsonization, which ultimately prolonged their circulation time in the body. The ET-NEs showed enhanced tumor growth inhibition, as compared to free ET after 24 h of incubation. Hence, ET-NEs lead to the advancement of a therapeutic approach against TNBC [295]. Miranda et al., 2021, developed lapachol-loaded nanoemulsion (LAP-NE) for the treatment of TNBC. The LAP-NE with 170 nm particle size showed increased internalization in tumor cells via EPR. Due to the nano-size, the NEs also escaped opsonization, which prolonged their circulation time. Moreover, the existence of negative zeta potential indicated the enhanced stability of the NE. Moreover, NEs exhibited a more sustained release, as compared to free drugs, thereby facilitating a controlled drug delivery system. Also, in comparison to free lapachol (IC_50_ = 6.60 ± 3.1 μM, relative tumor volume = 5.51), LAP-NE showed increased cytotoxicity (IC_50_ = 7.29 ± 1.79 μM) and reduced relative tumor volume (3.22), thereby providing an effective therapeutic strategy for TNBC therapy [296]. 

### 5.8. Solid Lipid Nanoparticles (SLN) 

SLNs are lipid-based nanocarriers comprised of solid lipid matrix and surfactants, with a diameter ranging between 50 and 1000 nm. They are considered less toxic and more biocompatible than polymeric and metallic nanoparticles. SLN offer advantages like high encapsulation of both hydrophilic and lipophilic drug, improved targeting, enhanced bioavailability, and feasibility of large-scale production [297,298,299]. Moreover, the heterogeneity within the molecular structure of SLN help in providing significant stability during its storage [300,301]. Wang et al., 2017, developed resveratrol-loaded SLNs (Res-SLN) for the treatment of TNBC. The Res-SLN showed smaller particle size (168.2 ± 10.7 nm.), negative zeta potential (−23.5 mV), and narrow size distribution (0.26), recommendable for enhanced physical stability, increased tumor site internalization, and targeted lymphatic uptake. Also, as compared to free resveratrol, the Res-SLNs exhibited a superior inhibitory activity over the growth, intravasation, and migration of TNBC cells. Hence, it was concluded that SLN provides a great platform for the treatment of TNBC [302]. Siddharta et al., 2018, fabricated di-allyl-disulfide loaded SLN, surface-functionalized with RAGE antibody (DADS-RAGE-SLN) to improve its bioavailability and increase its targetability to TNBC cells. Due to the presence of lipids, DADS-RAGE-SLN experienced flexible encapsulation, which resulted in sustained drug release. Moreover, due to the small size (148.18 ± 1.73 nm) and the presence of surface antibodies, the DADS-RAGE-SLN undergoes increased accumulation in the acidic tumor microenvironment. Also, compared to free DAD (15%), DAD-RAGE-SLN (61.8%) showed an enhanced cytotoxic effect over TNBC cells (MDA-MB231). Hence, RAGE functionalized SLN provides a promising strategy for improving the antitumor activity of cytotoxic drugs, without affecting the non-malignant cells [303]. Pindiprolu et al., 2018, fabricated niclosamide-loaded SLN (Niclo-SLN) for the effective treatment of TNBC. The lipidic core of SLN showed enhanced entrapment efficiency (82.21 ± 0.62%) and a smaller size (112.18 ± 1.73 nm) exhibited enhanced internalization within the tumor cells. The Niclo-SLN showed initial burst release in the initial hour followed by a sustained drug release. Moreover, more drug gets released in the acidic tumor microenvironment (90%) as compared to physiological pH (25%). Further, Niclo-SLN (70%) showed an increased apoptotic rate as compared to free Niclo (50%). Hence, it offered an approaching delivery system for the treatment of TNBC [304]. Eskiler et al., 2019, prepared talazoparib loaded SLNs for the treatment of *BRCA1* deficient TNBC. The presence of lipid in SLN created a stable core that facilitated improved entrapment efficiency (85%). Also, the talazoparib-SLN exhibited a sustained drug release profile (51%), as compared to free talazoparib (89%), which resulted in proper utilization of the drug. Moreover, talazoparib-SLN (85.56%) showed enhanced apoptosis to BRCA1 deficient TNBC cells (HCC1937-R cells), as compared to free talazoparib (25.86%) [305]. Rocha et al., 2020, developed docetaxel-loaded SLN (SLN-DTX) for preventing the growth of TNBC along with inhibition of lung metastasis. The small particle size of the SLN-DTX (128 ± 2.2 nm) indicated enhanced accumulation of SLN in tumor cells. The binding energy prevailed within the lipids and drugs mediated enhanced entrapment efficiency (86 ± 2.4%), thereby improving the solubility and stability of DTX. Moreover, the SLN-DTX showed a rapid release in the initial hours, followed by controlled release, resulting in effective treatment of TNBC, without having any lag treatment period. The SLN-DTX showed enhanced cytotoxicity (IC_50_ = 0.08 µg/mL), which further resulted in increased apoptosis, as compared to free DTX (IC_50_ = 10 µg/mL). Moreover, the histological studies of lungs showed that SLN-DTX exhibited decreased expression of IL-6 serum levels, ki-67, and BCL-2 expression, as compared to free DTX, and thereby indicated reduced lung metastasis [306]. Hence, it was concluded that SLN offered an encouraging drug delivery system for the effective treatment of TNBC and the prevention of metastasis. SLN also exhibits certain limitations like low drug loading capacity and the possibility of drug expulsion due to their perfect crystalline structure and the development of the crystallization process on storage, respectively. Additionally, SLN also facilitates the initial burst release of the drugs. It was revealed that for overcoming such limitations, the researchers have developed second-generation nanoparticles, named nanostructured lipid carriers (NLCs) [307].

### 5.9. Nanostructured Lipid Carriers (NLC)

NLC were introduced in the late 1990s and are considered second-generation lipid-based nanocarriers. NLC are composed of an amalgamation of solid lipid and liquid lipid along with surfactant [308]. NLC further overcome the disadvantages of SLN, which include poor drug loading and drug expulsion on storage due to their imperfect crystal structure and the addition of liquid lipid with solid lipid into the lipidic matrix [307] In addition, NLC also improve the drug solubility, enhances the site-specific internalization, undergoes lymphatic uptake thereby facilitating escape from RES, and offers a sustained drug release profile [309]. Godugu et al., 2016, prepared diindolylmethane (DIM) derivatives loaded NLC for increasing its bioavailability and effective treatment of TNBC. The small size of NLC showed enhanced internalization in tumor cells. Also, a significant increment in oral bioavailability (4.73-fold increase in Cmax; 11.19-fold increase in AUC) was observed in the case of NLC as compared to free DIM derivative. Moreover, the anticancer studies showed a significant decrease of tumor volume and tumor weight in the TNBC cell line when treated with DIM derivative loaded NLC, as compared to free DIM derivative [310]. Singh et al., 2017, developed lycopene-loaded NLC for efficient oral absorption and effective TNBC treatment. NLC showed enhanced tumor site accumulation due to their smaller size. Moreover, the lipidic core facilitated increased encapsulation that resulted in an improved entrapment efficiency. NLC exhibited a rapid release during initial hours followed by sustained release with a cumulative % drug release of 82.33 ± 3.67%. Further, the lycopene-loaded NLC showed enhanced cytotoxicity, as compared to free lycopene, thereby inducing enhanced apoptosis in TNBC cells [311]. Ong et al., 2018, developed thymoquinone (TQ) loaded NLC for enhanced anticancer activity against TNBC. NLC showed improved entrapment efficiency and drug loading, along with enhanced site-specific internalization via EPR. In addition, the TQ-NLC showed enhanced apoptosis and anti-metastatic effect as compared to free TQ. Hence, NLC provide an effective platform for the delivery of drugs to treat TNBC [312]. Kebebe et al., 2019, formulated gambogic acid (GA) loaded NLC, functionalized with dimeric c (RGD) to improve the anticancer activity for the TNBC therapy. GA-cRGD-NLC showed enhanced tumor site accumulation. Moreover, the GA showed enhanced encapsulation within the lipidic core, resulting in enhanced stability of GA within the nanocarrier. In addition, GA-cRGD-NLC (0.25 µg/mL) showed increased cytotoxicity, as compared to untargeted GA-NLC (0.5 µg/mL) [313]. Nordin et al., 2020, prepared citral-loaded NLC for the effective treatment of TNBC. The citral-NLC showed increased internalization in the tumor cells due to its small size and improved entrapment efficiency. Moreover, citral-NLC showed superiority in apoptosis, migration, invasion, and wound healing assay, as compared to free citral. Hence, NLC provided an effective system of drug delivery for the efficient treatment of TNBC [309].

The various nanoformulations fabricated in the last decade to enhance therapeutic efficacy over the conventional formulations in offsetting TNBC have been summarized in Table 3.

## 6. Nanomedicine: From Pre-Clinical Design to Clinical Practice

Nanomedicines are able to modulate the biodistribution of the drug as well as target them to the desired site, thereby improving the balance between efficacy and toxicity. In the pre-clinical studies, it was observed that nanoparticles inhibit the growth of cancer as well as prolong the survival rate as compared to the non-formulated drugs, however, in the clinical studies, the patients obtain benefit from nanoparticles only because of altered or decreased side effects. Hence, it leads to an increased number of preclinical studies compared to a smaller number of nanomedicines approved for clinical practice. Thus, to tackle the clinical challenges in the right way, a certain protocol was established [314]. 

It was observed that the national competent authority announced a clinical trial authorization, which stated that the content of the protocol and its format must comply with the Community guideline on Good Clinical Practice (CPMP/ICH/135/95). To administer any nanoparticle in humans, various steps have to be followed. The first step is the preparation of IMPD, which involves the compilation of all information associated with the drug substance (Part S), and the investigational medical product under test (Part P), where the drug substance can be natural, or synthetic, and the product is the nanoformulation of the drug. The second step is to complete the Investigator’s Brochure, which contained five chapters, assembling both clinical and non-clinical information relevant to the human subject. The first chapter includes a mini-review of the biological properties of the product (nanoformulation) and their effect on the human. The second chapter includes a summary of IMPD Part S and P. The third chapter involves the results of all pre-clinical analyses (in vitro and in vivo pharmacology; biodistribution, PK, dosimetry, and toxicity studies). The fourth chapter deals with a summary of all results and data previously obtained in humans with the administration of the investigated nanoformulation. The last chapter offers the investigators guidance in summarizing the information necessary for clinical practice (clinical indications, dose, administration route, contraindications, special warnings, and reference safety information). Finally, a study protocol was to be prepared, indicating the justification for the rationale, summary and procedure of the trial, eligibility criteria, the treatment regimen administered to the volunteers, and the efficacy assessment. In addition to these, the study protocol should also include information concerning the safety assessment, regulatory issues, quality control, legal consideration, and funding and insurance issues [315].

## 7. Toxicity of Nanoparticles

Nanoparticles are comprised of different types of materials with specific chemical structure, solubility, size, morphology, and surface charge, which may influence the toxicity of the nanoparticles [316]. It was further observed that the nanoparticles exhibit a certain level of toxicity due to their small size and increased surface area, as their nano dimension can lead to irreversible oxidative strain, protein denaturation, agitation of asthmatic and allergic reactions, etc. [317]. On further analysis of the nanoparticles, it was observed that the toxicity of the liposomes and dendrimers are mainly due to their small particle size and surface charge. For example, it was observed that the cationic liposomes interact with serum proteins, lipoproteins, and extracellular matrix, which leads to either aggregation or expulsion of drugs before entering the target site, which results in systemic toxicity. Also, dendrimers with a positive charge like amino-terminated PAMAM dendrimers destabilize the cell membrane, resulting in cell lysis. Such side effects could be overcome by optimizing the factors responsible for nanoparticle preparation, which in turn controls the size and surface functionalization, thereby modifying the surface charge. However, the polymeric nanoparticles seemed to exhibit less toxicity as the polymers used are biodegradable and are easily eliminated from the body with no residual materials. It was further observed that the toxicity of the nanoparticles is also associated with their composition. Due to the presence of silver, the silver nanoparticles generate ROS, which can destroy the blood-brain barrier and form neuronal degeneration and brain edema [318]. Also, there is evidence that states that the carbon nanotubes (both SWCNT and MWCNT) can induce oxidative stress and cytotoxicity [316].

Research evidence demonstrated that nanoparticles are also responsible for inducing allergic reactions. However, it was observed that nanoparticles induce allergic reactions by acting as adjuvant instead of hapten, which activates a certain type of cytokines, antibodies, and cells favoring allergic sensitization to environmental allergens [319]. From various studies, it was observed that lipid-based nanoparticles like liposomes, lipid micelles, nanoemulsion, SLN, and NLC can activate the complement system via classic and alternative pathways. Such activation further results in the development of complement activation-related pseudoallergy, an acute hypersensitivity. Hence, it was concluded that the factors responsible for activation of the complement system include surface charge (positive/negative), an increment in size from 70 nm to 300 nm, composition of lipid-based nanoparticles, and their amount in the formulation and functionalization of the surface with anionic PEG-PE. The common symptoms of complement activation-related pseudoallergy are shortness of breath, flushing, dyspnea, rash, chest and back pain, and distress [320].

## 8. Conclusions

TNBC remains an aggressive type of breast cancer with a poor prognosis due to the discrepancy in its molecular and genomic profiling. Although chemotherapy has been the backbone of TNBC treatment, certain developments have occurred with the targeted therapy. This has led to the emergence of two PARP inhibitors: olaparib and talazoparib, as approved by the FDA in 2018. However, these drugs were restricted to patients who suffered from BRCA1/2 mutation, which comprised only 10–15% of the TNBC population. However, a lot of recent advancements have been made in the advancement of novel nanocarrier-based delivery systems, which could offer a ray of hope in improving the treatment and health options among patients suffering from TNBC. Nanoparticles provide plenty of opportunities in improving the biopharmaceutical attributes of the drug, their bioavailability, and targetability, drug loading capacity, ability to administer drug combination, and limiting off-target toxicities and MDR due to their versatility in terms of morphology, surface characteristics, and material of their fabrication. In recent times, much focus has been given to the utilization of biocompatible and green technologies for the preparation of nanoparticles. The novel nanotechnologies are further amalgamated with various other disciplines like synthetic chemistry, molecular biology, formulation development, molecular imaging, etc., to develop a better understanding between the components of the nanoparticles and their association with the cellular environments in vivo. To conclude, there arises an expectation that nano-cavalries loaded with drugs, genes, or endogenous factors will soon become an element of the armamentarium for the treatment of TNBC and aid to manage the peril and commit to improved patient survival. 

## 9. Future Perspective

This review has discussed various types of nanoparticles optimized for the therapeutic delivery of drugs and to overcome the heterogenous physiological barriers found across the patients suffering from TNBC. It was observed that nanoparticles exist in many shapes and sizes, ranging from inorganic nanoparticles to polymer-based nanoparticles. It was further observed that a few of the polymer-based nanoparticles or inorganic nanoparticles showed significant in vivo results for clinical application. Our view suggests that such nanoparticles can induce certain toxicity in humans, which is not observed in the case of lipid-based nanoparticles, and urges the evaluation of the substantial preclinical studies with utmost caution. In our opinion, lipid-based nanoparticles, more specifically SLN and NLC, will make a substantial impact on the treatment and management of TNBC. 

## Figures and Tables

**Table 1:** Different classes of anticancer drugs and their suitability in different TNBC subtypes.

TNBC Subtypes	Therapeutic Approaches	Therapeutic Classes	Examples
Basal-like 1 (BL-1)	Inhibits cell division, and interfere with DNA responses	Taxanes	Paclitaxel, Docetaxel
		Platinum agents	Cisplatin, Carboplatin, Oxaliplatin
		Anthracyclines	Doxorubicin, Daunorubicin, Etoposide
		PARP inhibitors	Olaparib, Rucaparib, Talazoparib, Niraparib
Basal-like 2 (BL-2)	Inhibits signaling of EGFR, and MET	Platinum agents	Cisplatin, Carboplatin, Eptaplatin, and Oxaliplatin
		PARP inhibitors	Olaparib, Rucaparib, Talazoparib and Niraparib,
		Growth factor inhibitors	Erlotinib, Gefitinib, Afatinib, Cetuximab, Panitumumab, Bevacizumab, and Pertuzumab
		mTOR inhibitors	Rapamycin, Everolimus, and RapaLink-1
Immunomodulatory (IM)	Interferes or inhibits immune responses or signaling	Platinum agents	Cisplatin, Carboplatin, Eptaplatin Nedaplatin, and Oxaliplatin
		PARP inhibitors	Olaparib, Rucaparib, Talazoparib and Niraparib
		Immune checkpoint inhibitors	Ipilimumab, Nivolumab, Pembrolizumab, Cemiplimab, Atezolizumab, Avelumab and Durvalumab
Mesenchymal (M)	Inhibit signaling pathways including Notch, Wnt, IGFR1, PI3K/AKT/mTOR, TGFβ, EGFR, Src, and EMT	Growth factor inhibitors	Erlotinib, Gefitinib, Afatinib, Avitinib, lapatinib, Cetuximab, Panitumumab, Vandetanib, Bevacizumab, and Pertuzumab
		mTOR inhibitors	Rapamycin, Everolimus, and RapaLink-1
		Src inhibitors	Dasatinib, Bosutinib,
		PI3K inhibitors	Idelalisib, Alpelisib
		AKT inhibitor	Ipatasertib, and Capivasertib
Mesenchymal stem-like (MSL)	Inhibit Wnt, PI3K/mTOR, EGFR, MAPK, TGFβ, Src, and EMT	Growth Factor inhibitors	Erlotinib, Gefitinib, Afatinib, Osimertinib, lapatinib, Cetuximab, Panitumumab, Vandetanib, Bevacizumab, and Pertuzumab
		mTOR inhibitors	Rapamycin, Everolimus
		PI3K inhibitors	Idelalisib, Alpelisib
		MAPK inhibitors	Trametinib, Dabrafenib
		Scr inhibitors	Bosutinib, Dasatinib
Luminal Androgen Receptor (LAR)	Inhibit AR signaling, PI3K/AKT/mTOR, and MAPK signaling, and FOXA1 signaling	Nonsteroidal antiandrogens	Enzalutamide, bicalutamide, orteronel
		mTOR inhibitors	Rapamycin, Everolimus
		PI3K inhibitors	Idelalisib, Taselisib

**Table 2:** Various clinical trials of TNBC using combination therapy.

Clinical Trial Identifier	Treatment Regimen	Start Year/End Year	Stage of TNBC	Phase	Trial Status/Interim Results	References
NCT03101280	Rucaparib (PARP inhibitor) + Atezolizumab (PD-1 inhibitor)	2017/2020	Advanced TNBC	I	Completed	[189]
NCT03544125	Olaparib (PARP inhibitor) + Durvalumab (PD-1 inhibitor)	2018/2020	Metastatic TNBC	I	Completed	[190]
NCT02657889	Niraparib (PARP inhibitor) + Pembrolizumab (PD-1 inhibitor)	2016/2021	Advanced and metastatic TNBC	I/II	Active; ORR 29%	[191]
NCT03167619	Olaparib (PARP inhibitor) + Durvalumab (PD-1 inhibitor)	2017/2021	Advanced, platinum treated TNBC	II	Active	[192]
NCT03150576	Olaparib (PARP inhibitor) + platinum-based Chemotherapy (Carboplatin)	2017/2032	TNBC	II/III	Recruiting	[193]
NCT02789332	Olaparib (PARP inhibitor) + Paclitaxel (Chemotherapy)	2016/2020	Early TNBC	II	Completed –The pCR of the combination was found to be 55.1%, as compared to 48.6% with paclitaxel	[194]
NCT02032277	Veliparib (PARP inhibitor) + standard neoadjuvant therapy (Carboplatin + paclitaxel + Cyclophospha- mide/doxorubicin)	2014/2020	Early TNBC	II-III	Completed –The pCR of the combina-tion was found to be 53%	[195]
NCT04039230	Sacituzumab govitecan (antibody-drug conjugate) + talazoparib (PARP inhibitor)	2019/2024	Metastatic TNBC	I-II	Recruiting	[196]
NCT03720431	TTAC0001 (mAb targeting VEGFR 2) + Pembrolizumab (PD-1 inhibitor)	2018/2022	Metastatic TNBC	I	Active, not recruiting	[197]
NCT03243331	Gedatolisib (dual PI3K/mTOR inhibitor) + PTK7-ADC (antibody-drug conjugate)	2017/2020	Metastatic TNBC	I	Completed	[198]
NCT03394287	SHR1210 (Anti-PD1-inhibitor) + Apatinib (tyrosine kinase inhibitor)	2018/2020	Advanced TNBC	II	Completed	[199]
NCT02723877	PQR309 (dual PI3K/mTOR inhibitor) + Eribulin	2016/2018	TNBC	I/II	Completed	[200]
NCT02457910	Enzalutamide (Non-steroidal antiandrogen) + Taselisib (PI3K inhibitor)	2015/2020	AR+ metastatic TNBC	I/II	Active, not recruiting	[201]
NCT02423603	Paclitaxel + AZD5363 (AKT inhibitor)	2015/2020	Advanced/metastatic TNBC	II	Active, not recruiting	[202]
NCT02583542	AZD2014 (mTORC1/2 inhibitor) + selumetinib (kinase inhibitor)	2015/2020	Advanced TNBC	I/II	Active, not recruiting	[203]
NCT00733408	Nab-paclitaxel (Chemotherapy) + Erlotinib (EGFR TKI) + Bevacizumab (VGEF mAb)	2008/2018	Metastatic TNBC	II	Completed	[204]
NCT01097642	Ixabepilone (chemotherapy) + Cetuximab (EGFR mAb)	2010/2019	TNBC	II	Completed	[205]
NCT02605486	Bicalutamide (AR inhibitor) + Palbocilib (CDK4/6 Inhibitor)	2015/2022	AR+ metastatic TNBC	I/II	Active, not recruiting	[206]
NCT02513472	Eribulin Mesylate (microtubule inhibitor) + Pembrolizumab (PD-1 inhibitor)	2015/2020	Metastatic TNBC	I/II	Active, not recruiting	[207]
NCT02530489	Nab-Paclitaxel (Microtubule inhibitor) + Atezolizumab (PD-L1 inhibitor)	2015/2023	TNBC	II	Active, not recruiting	[208]
NCT02752685	Pembrolizumab (PD-L1 inhibitor) + Nab-paclitaxel (Microtubule inhibitor)	2016/2021	Metastatic TNBC	II	Recruiting	[209]
NCT02672475	Galunisertib (TGF-b inhibitor) + Paclitaxel (Microtubule inhibitor)	2016/2023	AR- metastatic TNBC	I	Active, not recruiting	[210]
NCT02456857	Liposomal Doxorubicin (intercalating agent) + Bevacizumab (VGEF mAb) + Everolimus (mTOR inhibitor)	2015/2022	Locally advanced TNBC	II	Active, not recruiting	[211]

**Table 3:** The various nanoformulations were fabricated to enhance the therapeutic efficacy over the conventional formulations in offsetting TNBC.

Nanoparticles	System	Observation	References
Liposomes	Dasatinib and Vincristine loaded liposomes	Dasatinib and vincristine-loaded liposomes exhibited targeted annihilation of VM channels by inhibiting VM indicators, resulting in the prevention of TNBC relapse. Further, the liposomes showed a delayed-release profile that enabled maximum drug delivery at the tumor site, facilitating maximum apoptosis with minimum leakage in circulation.	[237]
	F3 peptide targeted liposomes encapsulating β3 integrin siRNA	The β3 integrin siRNA-loaded liposomes silenced the expression of overexpressed β3 integrin in TNBC cells. Moreover, targeted liposomes showed no sign of metastasis and relapse even after 4 weeks of post-treatment, as compared to untreated cells.	[238]
	Cationic PEGylated liposomes loaded with gambogic acid (GA)	The liposomes showed >50% reduction of tumor volume and a 1.7-fold decrease in tumor weight when compared with GA alone.	[239]
	Irinotecan loaded liposomes	The irinotecan-loaded liposomes exhibited prolonged plasma drug exposure with 17.7 ± 3.8 h MRT, as compared to free irinotecan (3.67 ± 1.2 h). Further, irinotecan-loaded liposomes showed increased accumulation in the metastatic lesion, as compared to free irinotecan.	[227]
	DSPE-PEG2000-tLyp-1 peptide-functionalized liposomes encapsulated with miRNA	The spherical nanosized liposomes (120 nm) were effectively captured by the TNBC cells and were targeted to mitochondria where the miRNA silenced the expression of the slug gene and resulted in the inhibition of the TGF-β1/Smad pathway and invasiveness.	[240]
	Slug gene Paclitaxel and piperine co-loaded liposomes	The percent encapsulation of paclitaxel and piperine in the liposomes was found to be 31% and 73%, respectively. The targeted liposome showed increased cellular uptake and improved cytotoxicity profile, as compared to the non-targeted counterparts.	[241]
Dendrimers	Phosphorus and polyamidoamine dendrimer loaded with PLK1 siRNA	The dendrimers showed enhanced internalization in tumor cells due to their cationic nature, which favors their interaction with the tumor cell, as compared to the solution.	[243]
	Poly(amidoamine) dendrimer encapsulated with doxorubicin	The dendrimers showed increased internalization in the tumor cells, along with effective tumor growth inhibition and prolonged survival.	[244]
	PAMAM dendrimers loaded with methotrexate and D-glucose	The spherical nano-ranged dendrimers (∼30 nm) were taken up by the tumor cells through the EPR effect and showed 2-fold increased tumor cell internalization due to the presence of positive charge on their surface (13 to 19 mV), as well as glucose moiety. In addition, the dendrimers showed 20% less cell viability as compared to free methotrexate.	[245]
Polymeric micelles	Suberoylanilide hydroxamic acid (SAHA) and paclitaxel co-loaded hybrid micelle	The polymeric micelles showed a rapid release profile, indicating a rapid onset of action. Moreover, a synergistic effect was observed from hybrid micelle (IC_50_ = 0.52 μg/mL), as compared to non-micellar combination (IC_50_ = 3.071 μg/mL).	[251]
	Doxorubicin and docetaxel co-loaded poly (D, L-lactide-co-glycolide) based polymeric micelle	The drug-loaded micelles showed increased internalization and accumulation in the tumor site and offered an effective drug release profile in acidic organelles along with the synergistic anti-tumor activity.	[252]
	2-hydroxy-ethylacrylate (HEA) and 2-ethylhexylacrylate (EHA) copolymerized orlistat loaded micelle.	The polymeric micelle exhibited increased apoptosis, and decreased tumor volume, as compared to free orlistat.	[253]
	Aminoflavone (AF)-loaded EGFR targeted polymeric micelle	The small-sized polymeric unimolecular micelle showed effective cellular uptake by endocytosis in the presence of endosomal pH as compared to blood pH, concomitant with increased stability. The polymeric micelles also exhibited increased targetability and apoptosis as compared to free AF.	[254]
	Curcumin derivative RL71 loaded styrene-maleic acid (SMA)-based micelles	SMA-RL71-micelle showed an improved biodistribution and 16-fold increased drug accumulation in the tumor site as compared to free RL71. In addition, the SMA-RL71-micelle exhibited increased apoptosis with no cytotoxicity.	[255]
	Honokiol-loaded nanomicellar system	Honokiol-loaded nanomicelles exhibited increased absorption that resulted in increased oral bioavailability (Cmax = 4.06 fold; AUC= 6.26), as compared to 40 mg/kg free drug. In addition, the nanomicelles also showed a significant reduction in tumor volume and weight as compared to free drugs.	[256]
	Epirubicin (EPI)-loaded polymeric micelles functionalized with pH triggered moiety	The polymeric micelles underwent selective accumulation and penetration in primary tumors and vascularized axillary lymph node metastasis. The pH-triggered moiety further facilitated drug release in the acidic tumor microenvironment, sparing the healthy tissues.	[257]
	Halofuginone hydrobromide (HF) loaded TGPS polymeric micelles	Polymeric micelles showed a sustained drug release profile with excellent stability and biocompatibility in vivo. They also exhibited enhanced tumor growth inhibition (68.17%) in comparison to free drug.	[258]
Polymeric Nanoparticles	Paclitaxel loaded chitosan nanoparticles (PTX-CS-NP)	The hemolytic toxicity profile of PTX-CS-NP was found to be 4-fold less than the free PTX. PTX-CS-NP showed a sustained drug release profile where approximately 60% of the drug was released within 24 h. Furthermore, the IC_50_ of PTX-CS-NP and free PTX were found to be 9.36 ± 1.13 μM, and 14.755 ± 1.68, respectively.	[268]
	PLA-b-PEG nanoparticle loaded with erlotinib (Ei), and DOPA- doxorubicin (DOPA-Dox)	The drug release profile showed that approximately 80% of Ei was released within 4 h while only 20% of Dox was released up to 24 h of administration. Also, NPs initiated accumulation inside the tumor within 1 h of administration, which continued up to 24 h due to the EPR.	[269]
	RGD-conjugated polymer-lipid hybrid nanoparticles co-loaded with doxorubicin (DOX) and mitomycin C (MMC)	NPs exhibited a 31-fold decrement in the burden of lung metastases, concomitant with a 57% longer median survival time.	[270]
	mPEG-PLGA co-polymers based piperine-loaded nanoparticle	The polymeric nanoparticles undergo passive diffusion into the tumor site without affecting the kidney, liver, and spleen. The piperine-loaded polymeric NPs inhibited the growth of TNBC and induced apoptosis while sparing normal fibroblast.	[271]
	PLGA-TPGS NPs loaded with quercetin	Polymeric NPs exhibited a sustained drug release profile, as compared to free quercetin. In addition, NPs inhibited the growth of TNBC and induced apoptosis while sparing normal fibroblast, as supported by an increased tumor inhibition ratio of 67.88%, along with fewer lung metastasis colonies. Furthermore, NPs provided an inhibitory effect upon the migration of uPA (Urokinase-type plasminogen activator) knockdown on TNBC cells.	[272]
Metallic Nanoparticles	Gold nanoparticles (AuNM) functionalized with a tyrosine kinase inhibitor, ZD6474	AuNM exhibited a slow and sustained release of ZD6474 (82% following 45 h) at pH 5.5. AuMN showed targeting due to their low cytotoxicity and immunogenicity. Moreover, AuNM inhibited tumor growth and prevented metastasis without causing any haemotoxicity.	[281]
	Curcumin loaded metal-organic framework (NMOF-3) tagged by folic acid (IRMOF-3@CCM@FA)	IRMOF-3@CCM@FA showed 55% drug release in pH 5.5, as compared to physiological pH (31%). Further, IRMOF-3@CCM@FA showed enhance apoptosis and targeted delivery of curcumin, as compared to free curcumin.	[282]
	Silver nanoparticles (AgNPs)	AgNPs exhibited accumulation to both TNBC and non-malignant breast cells, but facilitate rapid degradation only in TNBC cells. Moreover, the internalization of AgNPs within the TNBC showed depletion of cellular antioxidants, causing endoplasmic reticulum stress and apoptosis.	[283]
Nanoemulsions	Omega 3-fatty acid derivative loaded nanoemulsion (NE)	The NE showed 99.9 ± 2.3% entrapment efficiency, enhanced tumor cell accumulation and a 50% reduced tumor weight as compared to the free derivative of omega 3 fatty acids.	[292]
	Doxorubicin hydrochloride and LyP-1 co-loaded self-micro-emulsifying drug delivery system (SMEDDS)	SMEDDS exhibited lymphatic uptake, thereby increasing bioavailability. Moreover, the SMEDDS showed enhanced in vivo cytotoxicity in p32 expressing TNBC cells, along with reduced tumor growth and metastasis.	[293]
	Decitabine (DAC) and panobinostat (PAN) co-loaded lipid nanoemulsion (LNEs)	LNEs showed increased stability, enhanced internalization in tumor cells, without affective liver, and spleen, and a controlled-release delivery system. Furthermore, LNEs showed increased inhibition of tumor growth of M subtype TNBC.	[294]
	Edelfosine nanoemulsions (ET-NEs)	ET-NE exhibited an enhanced passive targeting into the tumor site via the EPR effect, with fewer chances of opsonization, and prolonged circulation time in the body. ET-NE also showed enhanced tumor growth inhibition, as compared to free ET.	[295]
	Lapachol—loaded nanoemulsion (LAP-NE)	LAP-NE showed increased internalization in tumor cells via EPR, with enhanced stability. NEs exhibited a sustained release profile, as compared to free drugs. Moreover, in comparison to free lapachol (IC_50_ = 6.60 ± 3.1 μM, relative tumor volume = 5.51), LAP-NE showed increased cytotoxicity (IC_50_ = 7.29 ± 1.79 μM) and reduced relative tumor volume (3.22).	[296]
SLNs	Resveratrol-loaded SLNs (Res-SLN)	SLN showed enhanced physical stability, increased tumor site internalization, and targeted lymphatic uptake. Also, Res-SLNs exhibited a superior inhibitory activity over the growth, invasion, and migration of MDA-MB-231 cells	[302]
	Di-allyl-disulfide loaded SLN, surface-functionalized with RAGE antibody (DADS-RAGE-SLN)	DADS-RAGE-SLN experienced flexible encapsulation, which resulted in sustained drug release. SLN showed increased accumulation in the acidic tumor microenvironment, and in comparison, to free DAD (15%), DAD-RAGE-SLN (61.8%) showed enhanced cytotoxic effect over TNBC cells (MDA-MB231).	[303]
	Niclosamide loaded SLN (Niclo-SLN)	SLN showed enhanced entrapment efficiency, with an enhanced internalization within tumor cells. The SLN showed initial burst release followed by sustained drug release. Further, Niclo-SLN (70%) showed an increased apoptotic rate as compared to free Niclo (50%).	[304]
	Talazoparib loaded SLNs	SLNs showed improved entrapment efficiency (85%). The SLN exhibited a sustained drug release profile (51%), as compared to free talazoparib (89%). Moreover, talazoparib-SLN (85.56%) showed enhanced apoptosis to BRCA1 deficient TNBC cells (HCC1937-R cells), as compared to free talazoparib (25.86%).	[305]
	Docetaxel loaded SLN (SLN-DTX)	SLN showed enhanced accumulation in tumor cells. The SLN-DTX showed an initial burst effect release, followed by controlled release. SLN showed enhanced cytotoxicity (IC_50_ = 0.08 µg/mL) as compared to free DTX (IC_50_ = 10 µg/mL). Further, SLN showed reduced lung metastasis as compared to free DTX.	[306]
NLCs	Diindolylmethane (DIM) derivatives loaded NLC	The NLC showed enhanced internalization in tumor cells, a significant increment in oral bioavailability (4.73-fold increase in Cmax; 11.19-fold increase in AUC), and a decrease of tumor volume and tumor weight in an MDA-MB-231 cell line, as compared to free DIM derivative.	[310]
	Lycopene loaded NLC	The NLC showed enhanced tumor site accumulation and increased encapsulation. Moreover, the NLC exhibited an initial burst release followed by sustained release with cumulative % drug release of 82.33 ± 3.67%. The lycopene-loaded NLC also exhibited enhanced cytotoxicity, as compared to free lycopene.	[311]
	Thymoquinone (TQ) loaded NLC	NLC showed improved entrapment efficiency and drug loading, along with enhanced site-specific internalization via EPR. In addition, the TQ-NLC showed enhanced apoptosis and anti-metastatic effect as compared to free TQ.	[312]
	Gambogic acid (GA) loaded NLC, functionalized with dimeric c (RGD)	GA-cRGD-NLC showed enhanced tumor site accumulation, enhanced encapsulation along with improved stability. In addition, GA-cRGD-NLC (0.25 µg/mL) showed increased cytotoxicity, as compared to untargeted GA-NLC (0.5 µg/mL)	[313]
	Citral loaded NLC	Citral-NLC showed increased internalization in the tumor cells and improved entrapment efficiency. Moreover, citral-NLC showed superiority in apoptosis, migration, invasion, and wound healing assay, as compared to free citral.	[309]

## Data Availability

Not applicable.
